# Single cell transcriptomics identifies stem cell-derived graft composition in a model of Parkinson’s disease

**DOI:** 10.1038/s41467-020-16225-5

**Published:** 2020-05-15

**Authors:** Katarína Tiklová, Sara Nolbrant, Alessandro Fiorenzano, Åsa K. Björklund, Yogita Sharma, Andreas Heuer, Linda Gillberg, Deirdre B. Hoban, Tiago Cardoso, Andrew F. Adler, Marcella Birtele, Hilda Lundén-Miguel, Nikolaos Volakakis, Agnete Kirkeby, Thomas Perlmann, Malin Parmar

**Affiliations:** 1 Ludwig Institute for Cancer Research, Box 240, SE-171 77 Stockholm, Sweden; 2 Department of Cell and Molecular Biology, Karolinska Institutet, SE-171 77 Stockholm, Sweden; 3 Developmental and Regenerative Neurobiology, Wallenberg Neuroscience Center, and Lund Stem Cell Centre, Department of Experimental Medical Science, Lund University, 22184 Lund, Sweden; 4 Present Address: Department of Cell and Molecular Biology, National Bioinformatics Infrastructure Sweden, Science for Life Laboratory, Uppsala University, Husargatan 3, SE-752 37 Uppsala, Sweden; 5 Novo Nordisk Foundation Center for Stem Cell Biology (DanStem), University of Copenhagen, 2200 Copenhagen, Denmark

**Keywords:** Embryonic stem cells, Embryonic stem cells, Regeneration

## Abstract

Cell replacement is a long-standing and realistic goal for the treatment of Parkinsonʼs disease (PD). Cells for transplantation can be obtained from fetal brain tissue or from stem cells. However, after transplantation, dopamine (DA) neurons are seen to be a minor component of grafts, and it has remained difficult to determine the identity of other cell types. Here, we report analysis by single-cell RNA sequencing (scRNA-seq) combined with comprehensive histological analyses to characterize intracerebral grafts from human embryonic stem cells (hESCs) and fetal tissue after functional maturation in a pre-clinical rat PD model. We show that neurons and astrocytes are major components in both fetal and stem cell-derived grafts. Additionally, we identify a cell type closely resembling a class of recently identified perivascular-like cells in stem cell-derived grafts. Thus, this study uncovers previously unknown cellular diversity in a clinically relevant cell replacement PD model.

## Abstract (web summary)

What happens to cells on engrafting into the brain in animal models to treat Parkinson’s disease is unclear. Here, the authors use scRNA-seq to examine ventral midbrain (VM)-patterned human embryonic stem cells after functional maturation in a pre-clinical rat model for Parkinson’s disease and identify perivascular-like cells.

## Introduction

Authentic and functional midbrain dopamine (DA) neurons and their progenitors can now be generated from human pluripotent stem cells (hPSCs) via a floor plate intermediate^1,2^. These cell preparations are both safe and functional when transplanted to animal models of Parkinson’s disease (PD)^3^. However, although resulting grafts from hPSCs contain large numbers of desired DA neurons, these therapeutic cells are a minor component of the grafts which is the case also for transplants from fetal brain tissue. A comprehensive understanding of the cellular composition of the graft has remained difficult to achieve due to limitations in histological methods that rely on pre-conceived notions concerning the cell types likely to be present in the grafts.

Here, we use single-cell RNA sequencing (scRNA-seq) combined with histological analyses to characterize intracerebral grafts from ventral midbrain (VM)-patterned human embryonic stem cells (hESCs) and VM fetal tissue after long-term survival and functional maturation in a pre-clinical rat model of PD. The analyses shows that while both cell preparations gave rise to neurons and astrocytes, oligodendrocytes is only detected in grafts of fetal tissue. On the other hand, a cell type closely resembling a class of newly identified perivascular-like cells is identified as a previously unknown component of hESC-derived grafts. We also confirm the presence of these cells in transplants from three different hESC lines, as well as from iPSCs. Thus, these experiments address an outstanding question in the field of cell replacement in neurological disease by identifying graft composition of hESC- and fetal cell-derived grafts. These results have important implications when designing clinical trials.

## Results

### scRNA-seq identifies composition of fetal VM and hESC-derived neural graft

**Fig. 1:**
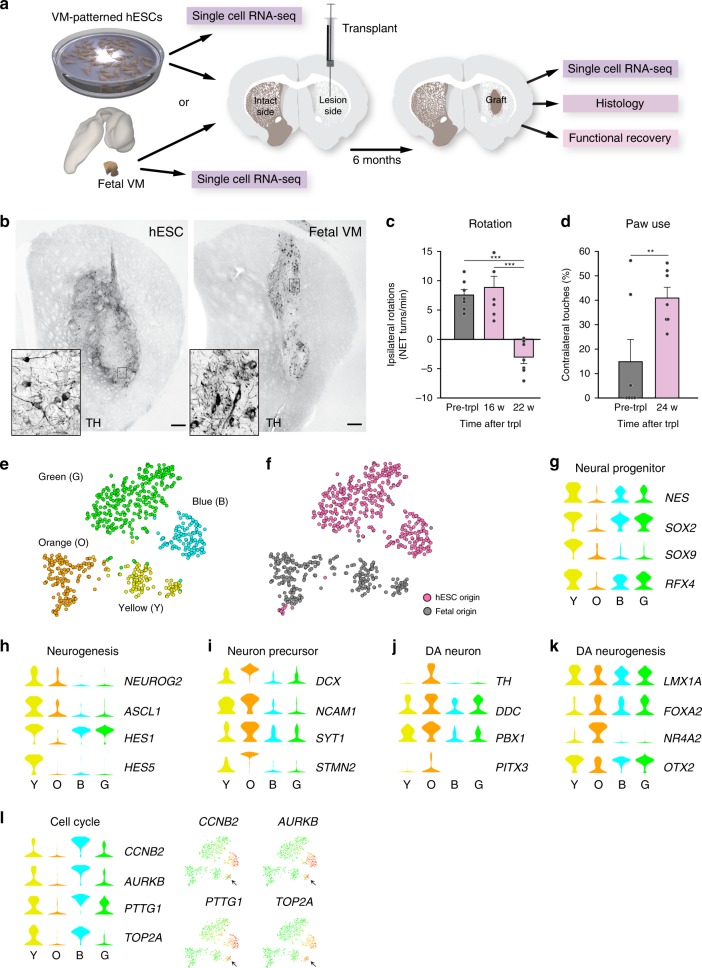
Histological validation and scRNA-seq analysis of progenitor cells before grafting in a pre-clinical cell therapy model of PD. **a** Schematic overview of experimental design. VM-patterned hESCs were grafted to seven rats and used as follows: scRNA-seq *n* = 2; histology *n* = 3; functional recovery *n* = 7. Cells from one fetal VM were grafted to three rats and used as follows: scRNA-seq *n* = 2; histology *n* = 1 (*n* = 1 because of limited access of fetal tissue). **b** Immunohistochemistry of TH in the graft core of hESC- and fetal VM-derived intrastriatal grafts 6 months post-transplantation. Insets show high-power magnifications of the DA neurons **c**, **d** Functional recovery of the hESC-derived cells by amphetamine-induced rotation test and spontaneous paw use (Cylinder) test (*n* = 7 rats; mean ± SEM; **p < 0.01, ***p < 0.001; compared to post-lesion; two-tailed paired *t*-test). **e** t-SNE showing clustering of 660 analyzed cells before grafting (404 cells of hESC origin, 256 cells of fetal origin). Green, blue, orange, and yellow circles define the clusters. **f** Same t-SNE as in **e** but with origin of cells marked with pink circles (hESC) or gray circles (fetal) as indicated. **g**–**l** Expression level per cluster for indicated genes. Genes represent markers for the cell types (neural progenitor, neuron precursor, DA neuron) or indicated processes (cell cycle, neurogenesis, or DA neurogenesis; see text for details). Expression levels of indicated cell cycle genes are also shown in the t-SNE. Scale bar, 250 µM.

To compare the developmental potential of hESC-derived VM progenitors with fetal VM cells after transplantation in a rat model of PD, hESCs were subjected to VM-patterning by a well-established protocol intended for generation of clinical-grade cell preparations^4^ and human fetal tissue was dissociated from the VM of a 7.5-week-old human embryo by the same protocol used for the fetal cell clinical transplantation trial, TRANSEURO^5^. Both types of cells were transplanted into the striatum of adult rats that had been unilaterally lesioned with 6-hydroxydopamine (6-OHDA) (Fig. 1a). Both fetal and hESC-derived cell preparations gave rise to neuron-rich grafts (Supplementary Fig. 1a, d) with innervation extending from the graft core to dorsolateral striatum (Supplementary Fig. 1b, e) and prefrontal cortex (Supplementary Fig. 1c, f). The grafts from both sources also contained the expected component of DA neurons as detected by the expression of tyrosine hydroxylase (TH) 6 months following transplantation (Fig. 1b). In agreement with previous observations^6^, TH-expressing DA neurons with mature morphology were found concentrated in the periphery of the grafts (Fig. 1b, insets). In addition, paw use and rotational asymmetry induced by 6-OHDA lesioning was corrected in animals transplanted with hESC-derived VM progenitors, confirming functional maturation after transplantation (Fig. 1c, d).

Cells were sampled for scRNA-seq both before grafting and 6 months after transplantation as illustrated in Fig. 1a. To separate intact cells from cell fragments and debris, fetal and hESC-derived progenitors were isolated by fluorescence-activated cell sorting (FACS) based on cell size (forward and side scatter) before grafting (Supplementary Fig. 1g). t-Distributed neighbor embedding (t-SNE) and graph-based clustering of the scRNA-seq data from fetal and hESC-derived cells before grafting resulted in four major clusters (green, blue, orange, and yellow in Fig. 1e). The two top clusters (green, blue) consisted almost exclusively of VM-patterned hESCs while the two bottom clusters (orange, yellow) consisted mostly of fetal cells (Fig. 1f), suggesting that while both fetal- and hESC-derived cells generate DA neurons with similar subtype specificities and functional properties after grafting^7,8^, the two different sources of cells were transcriptionally distinct at the time of transplantation.

SAMseq was used to identify genes enriched in each cluster (Supplementary Data 1, Supplementary Fig. 2a, b). Neural progenitor/radial glial cell markers including *NES*, *SOX2*, *SOX9,* and *RFX4* were prominently expressed in the yellow fetal cell-dominated cluster as well as in the blue and green hESC-dominated clusters consistent with the expression of these genes under DA neurogenesis in mouse and humans^9,10^ (Fig. 1g, Supplementary Fig. 2c). Regulators of neurogenesis (*NEUROG2*, *ASCL1*, *HES1*, and *HES5*) were prominently expressed in the yellow fetal cell-dominated cluster while the blue and green hESC-dominated clusters mainly expressed *HES1* (refs. ^9,10^) (Fig. 1h, Supplementary Fig. 2d). Importantly, transcription factors such as *LMX1A*, *FOXA2*, and *OTX2* that are critical in VM development and DA neurogenesis, as well as genes that are predictive of successful graft outcome (*EN1* and *DLK1*)^11,12^, were expressed by both fetal- and hESC-derived cells (Fig. 1k, Supplementary Fig. 2e). Cells in the fetal cell-dominated orange cluster (Fig. 1e) expressed genes that are normally active in early developing neurons including *DCX*, *NCAM1, SYT1*, and *STMN2*, as well as genes that are normally expressed in maturing DA neurons including *NR4A2, TH*, *DDC*, *PITX3*, and *PBX1* (Fig. 1i–k, Supplementary Fig. 2e–g). Of note, cells expressing markers for more mature DA neurons could be distinguished in one part of the orange cluster, thus indicating neuronal diversity among more mature fetal cells (Supplementary Fig. 2g). Markers of pluripotency (*POU5F1, NANOG*), mesoderm (*T*) or endoderm (*SOX17*) development were not expressed in any of the cell clusters (Supplementary Fig. 2h, i).

Cell cycle scores were predicted based on a list of known cell cycle genes and cells assigned to different cell cycle phases, which indicated the presence of cycling cells within the blue (hESCs) and yellow (fetal) clusters (Fig. 1l, Supplementary Fig. 3a). From the list of enriched genes, it is also evident that the main difference between the green and blue clusters is expression of cell cycle genes in the blue cluster (Supplementary Data 1). To assess if clustering was critically influenced by cell cycle, the cell cycle scores were regressed out and included in an independent clustering experiment. This clearly shows that cell cycle genes are the distinguishing feature of the blue and green clusters (and also drives sub-clustering of the yellow (fetal) cluster) (Supplementary Fig. 2j, k). However, no additional diversity of other cell clusters became evident when cell cycle genes were omitted from the analysis.

Six months after transplantation, when grafts had reached functional maturity, the transplants were dissected from the striatum, dissociated into a single-cell suspension, and subjected to FACS (see Fig. 1a, Supplementary Fig. 1h). The transplanted hESCs contained a GFP-encoding transgene expressed in virtually all transplanted cells (Supplementary Fig. 1k–m). Fetal cells did not carry a GFP-encoding transgene and were therefore isolated by FACS based on cell size (forward and side scatter) to separate single cells from debris (Fig. 1a, Supplementary Fig. 1g). Notably, since fetal cells did not express GFP, the vast majority of single cells isolated from fetal grafts were contaminating rat cells and scRNA-seq data from fetal transplants was therefore limited to only 63 fetal cells. In contrast, several hundred cells in each condition could be analyzed from hESC-derived grafted cells as well as from both fetal- and hESC-derived cells before grafting (Supplementary Fig. 1j).

**Fig. 2:**
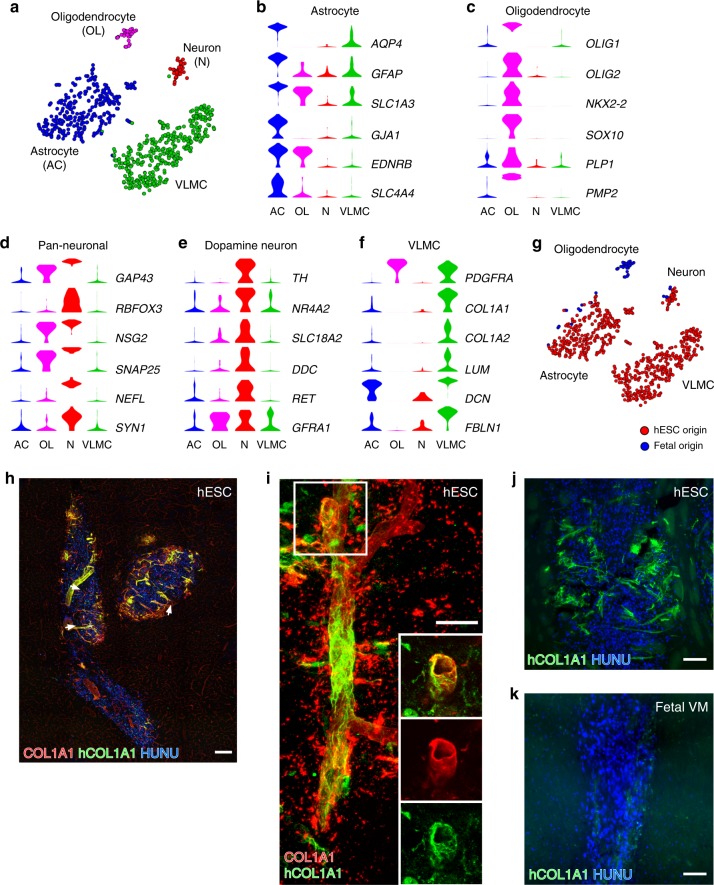
scRNA-seq analysis and histological validation of grafted cells into the striatum. **a** t-SNE showing clustering of 746 cells grafted to striatum (683 cells of hESC origin, grafted rats *n* = 2; 63 cells of fetal origin, grafted rats *n* = 2). Cell type assignments are indicated: OL oligodendrocyte, N Neuron, AC astrocyte, VLMC vascular leptomeningeal cells. **b**–**d** Expression level per cluster for indicated genes. All indicated genes are significantly enriched and established markers for astrocytes, oligodendrocytes, and pan-neuronal cells, respectively. **e** Expression level per cluster for selected dopaminergic markers. **f** Expression level per cluster for genes that are significantly enriched in the VLMC cluster and established markers for barrier-forming fibroblasts including VLMCs. **g** t-SNE of grafted cells as shown in Fig. 2a. Cells are marked according to their origin from either hESC-derived (red circles) or fetal-derived (blue circles) transplants. **h** Staining using antibodies recognizing both rat and human COL1A1 or only hCOL1A1 as indicated in the core of a hESC-derived graft from the same experiment as used for scRNA-seq. Human nuclei were counterstained with HUNU. **i** Representative immunofluorescence micrograph of hCOL1A1-positive cells intermingled with host-derived COL1A1-positive cells in close association with blood vessels. Boxed area shows the localization of the close-ups. **j**–**k** Representative micrographs of hCOL1A1/HUNU immunostaining from a hESC-derived graft (**j**) and from a fetal-derived grafts from the same experiment as used for scRNA-seq. Scale bars, 200 µM (**h**), 20 µM (**i**), and 100 µM (**j**, **k**).

Using graph-based clustering, fetal- and hESC-derived cells sampled from the rat striatum six months after grafting segregated into four main clusters (Fig. 2a). We used SAMseq^13^ for the identification of genes enriched in each cluster (see Methods; Supplementary Data 2, Supplementary Fig. 4b). From inspection of the most highly enriched genes in each cluster it was evident that three of the clusters contained cells expressing genes characteristic of astrocytes (*AQP4* and *GFAP)*, oligodendrocytes (*OLIG1* and *OLIG2*), and neurons (*GAP43* and *RBFOX3)*, respectively (Fig. 2b–d, Supplementary Fig. 4c–e). Of note, *PMP2*, one of the most significantly enriched genes in the oligodendrocyte cluster, is a marker for peripheral Schwann cells in mouse but was recently shown to be prominently expressed in human oligodendrocytes^14^. In addition, dopaminergic markers including *TH*, *SLC18A2*, and *DDC* were expressed in cells in the neuron cluster indicating, as expected, a high proportion of DA neurons (Fig. 2e, Supplementary Fig. 4f). Further analyses of publicly available datasets reporting both bulk and scRNA sequencing provided additional strong support for the assignment into astrocytes, oligodendrocytes, and neurons^14–17^ (Supplementary Fig. 5a). Of note, despite transplantation of the cells at a highly proliferative stage, only a very small number of cells (1.3%) showed cell cycle scores indicative of some cycling cells after 6 months in vivo, and these cells all belonged to the oligodendrocyte and astrocyte clusters (Supplementary Fig. 3b).

It is notable that the overall proportion of neurons relative to other cell types was low (<10%) in the scRNA-seq analysis from both fetal- and hESC-derived grafts (Fig. 2a). This finding significantly diverged from the high neuronal content observed in previous histological analyses of rats grafted with VM-patterned hESCs^2,11,18–20^. We therefore further investigated the neuronal content in brain sections of animals grafted at the same time with the same preparation of fetal or hESCs cells that were used for sequencing. In contrast to the scRNA-seq data, immunohistochemical analysis (Supplementary Fig 1a, d, Supplementary Fig. 5b) and quantifications of NeuN+ cells (Supplementary Fig. 5c) indicated a neuronal content comparable to that seen in previously analyzed grafts^2,8,11^ (Supplementary Fig. 5c). The apparent underrepresentation of neurons among recovered cells in the scRNA-seq data was not surprising considering difficulties to achieve complete mechanical dissociation of brain tissue into single-cell suspensions of mature neurons containing extensive projections^15,21^. Our data therefore underscore the importance of validating both qualitative and quantitative results from scRNA-seq datasets by alternative methods.

### Vascular leptomeningeal cells are a previously unknown component in hESC-derived grafts

The scRNA-seq data also identified a fourth cluster of cells which could not, based on the top enriched genes, be easily assigned to a known neural cell type (Supplementary Data 2, Supplementary Fig. 4b). Gene enrichment analysis using the top distinguishing genes of this cluster identified highly significant biological functions associated with vasculature and connective tissue, including genes encoding collagens and other extracellular components (Fig. 2f, Supplementary Fig. 6a, Supplementary Data 3). We then probed a large scRNA-seq dataset from the mouse brain interrogating more than 400,000 cells organized into 250 annotated clusters representing diverse cell types such as central and peripheral neurons, astrocytes, oligodendrocytes, Schwann cells, immune cells, and brain vascular cell types^15^ (Mouse Brain Atlas, http://mousebrain.org/). When this dataset was searched for cell types co-expressing enriched genes of the fourth cluster, “enteric glia” and “vascular leptomeningeal cells” (VLMCs) were identified as cell type categories with highly related gene expression profiles (Supplementary Fig. 6b, c). Enteric mesothelial fibroblasts, the closest matching cell type among “enteric glia”, and VLMCs belong to a family of highly related organ barrier-forming fibroblast populations^15^. Recent scRNA-seq analyses have identified related cells associated with the mouse brain vasculature^16,22^, characterized by the co-expression of *Pdgfra*, *Col1a1*, *Col1a2*, *Lum*, and *Dcn*^15,16,22^. Additionally, the human homologs of these genes were all expressed in the unknown cluster (Fig. 2f, Supplementary Fig. 4g). Therefore, we refer to these cells as VLMCs as this cluster consists of cells with striking transcriptional resemblance to previously described populations of barrier-forming fibroblasts, including VLMCs of the mouse central nervous system^15,16,22^.

The analysis of graft composition uniquely allowed us to discern similarities and potential differences between the cellular composition of fetal- and hESC-derived grafts after transplantation. Strikingly, tracing the origin of grafted cells visualized by t-SNE suggested prominent differences in generated cell types (Fig. 2g). Accordingly, two of the clusters (astrocyte and neuron clusters) contained cells both from grafted VM-patterned hESCs and from fetal cells. In contrast, the oligodendrocyte cluster contained only fetal-derived cells while the VLMC cluster contained only hESC-derived cells (Fig. 2g). The scRNA-seq analysis of cells after grafting thus suggested that VM-patterned hESCs and fetal VM cells have distinct developmental potential after grafting. Based on the unexpected identification of VLMCs in the hESC grafts, we examined highly expressed genes found in the VLMC cluster (*COL3A1*, *FBLN1*, *S100A11*, and *IFITM2)*, and additional genes encoding components of the extracellular space (e.g. *EMP2* and *MMP2*), also in the cells prior to grafting (Supplementary Fig. 6d). We found that several of these VLMC-associated genes were expressed together with DA progenitor markers in the same hESC-derived cells suggesting that a common hESC-derived precursor has potential to generate neurons, astrocytes, and VLMCs. t-SNE and graph-based clustering including all sequenced cells before and after grafting resolved the same clusters as when cells were analyzed separately (Supplementary Fig. 6e, f).

To validate the key observations from the scRNA-seq analysis of transplanted cells, and to investigate in more detail the properties of graft-derived VLMCs, we first confirmed the presence of VLMCs by histological analysis of brain sections of animals grafted in parallel with the same batches of cells that were used for scRNA-seq. Using antibodies recognizing both rodent and human COL1A1 (uniquely expressed in the VLMC cluster among the transplanted cells), and an antibody that is specific for the human protein (hCOL1A1) we confirmed the presence of VLMCs of human origin in the hESC-derived grafts (Fig. 2h). These COL1A1^+^ cells in the hESC-derived grafts were commonly located in close association with blood vessels penetrating into grafts (arrows in Fig. 2h, and high magnification in Fig. 2i), which is consistent with previous studies describing VLMCs associated with the brain vasculature^15,16,22^. The histological analysis also indicated that the hESC-derived VLMCs were intermingled with rat cells of similar appearance and location within the transplants (Fig. 2h, i, j), suggesting that cells originating from both host and graft contributed to the vascularization as previously reported for rodent-to-rodent neural transplants^23,24^. Only weak and non-distinctive signal was seen in transplants originating from the fetal cells grafted at the same time as the cells used for sequencing (Fig. 2k). In addition, and in line with findings obtained from the scRNA-seq dataset, histological analysis also confirmed the presence of graft-derived oligodendrocyte progenitors expressing OLIG2 and PDGFRA at the edge of fetal VM grafts (Supplementary Fig. 7c–e) but not in hESC-derived grafts (Supplementary Fig. 7f). To further assess the observed absence of fetal VM-derived VLMCs, we established primary cultures from two more embryos (gestational age 7.5 and 9 weeks). Both embryos gave rise to cultures rich in TH neurons in vitro, but no COL1A1-expressing cells were detected in the cultures (Supplementary Fig. 7a, b). Thus, although it is impossible to exclude the presence of VLMCs in fetal grafts, the immunostaining of cells in vitro and after transplantation in vivo are consistent with the findings from scRNA-seq suggesting different potential to generate VLMCs and oligodendrocytes depending on the origin of the transplanted cells (Fig. 2g).

**Fig. 3:**
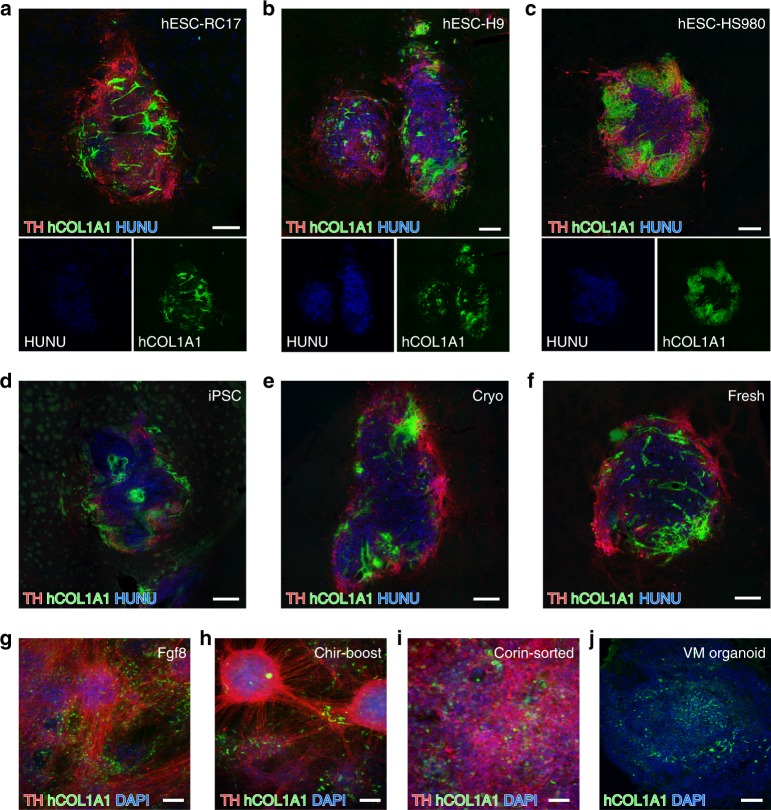
Presence of VLMCs in different cell lines. **a**–**c** Representative immunofluorescence micrograph showing staining of hCOL1A1 in TH-rich hESC-derived grafts from three additional grafting experiments where the transplant was generated from VM-patterned RC17 (**a**), H9 (**b**)^25^, and HS980 hESC lines (**c**)^4^. **d** TH/hCOL1A1 immunofluorescence staining of a VM-patterned iPSC-transplant sorted for IAP expression prior to transplantation^25^. **e**, **f** TH**/**hCOL1A1 immunofluorescence staining of VM-patterned hESC-derived grafts generated from cryopreserved cells (**e**) or fresh cells (**f)**. **g**–**i** Representative micrographs of TH/hCOL1A1 double immunostaining in terminally differentiated hESC in vitro cultures derived by three different clinically relevant VM-patterning differentiation protocols: the protocol used in this study (**g**), a protocol developed in the Studer lab that uses CHIR boost instead of FGF8 for proper caudalization (described in https://patents.justia.com/patent/20180094242) (**h**), and a protocol developed by the Takahashi lab where the cells are sorted based on Corin prior to grafting^12,26^ (**i**)). Nuclei were counterstained with DAPI in all three cultures. **j** hCOL1A immunostaining in a self-organized midbrain patterned organoid. Scale bars, 200 µM (**a**–**f**) and 100 µM (**g**–**j**).

To assess if VLMCs are also found in DAergic grafts derived from other pluripotent stem cell lines and when using other differentiation protocols, we next assessed the presence of VLMCs in grafts from previous transplantation experiments using VM-patterned progenitors derived from three different hESC lines, RC17 (differentiated using a GMP protocol showing minimal variability^4^), H9 (differentiated using a research grade protocol), and HS980 (refs. ^4,7,8,11^) (differentiated using a GMP protocol) (Fig. 3a–c), and an iPSC line (differentiated using a GMP protocol and including a MACS purification step prior to grafting (Fig. 3d)^25^. Importantly, graft-derived hCOL1A1^+^ cells were present in all analyzed VM-patterned hPSC-derived grafts independent of cell line or differentiation protocol (Fig. 3a–d). Clinical delivery will be done with cells that have been cryopreserved prior to transplantation, and VLMCs were shown to be present to a similar extent in grafts from fresh and cryopreserved cells (Fig. 3e, f).

To investigate whether the derivation of VLMCs was a characteristic uniquely associated with the specific differentiation protocols used in this study, we assessed hESCs after terminal differentiation in vitro using our standard protocol (Fig. 3g, Supplementary Fig. 7g) and two additional recently published protocols for generating VM progenitors for clinical use from hPSCs^12,26^; see also (https://patents.justia.com/patent/20180094242) (Fig. 3h, i, Supplementary Fig. 7h, i) as well as in three-dimensional self-organizing organoids (Fig. 3j, Supplementary Fig. 7j). These protocols all gave rise to cultures that are rich in TH^+^ neurons and cells co-expressing the VLMC markers COL1A1 and PDGFRA (Fig. 3g–j, Supplementary Fig. 7g–j).

### Graft placement does not affect cellular composition of VM-patterned hESC-derived grafts

**Fig. 4:**
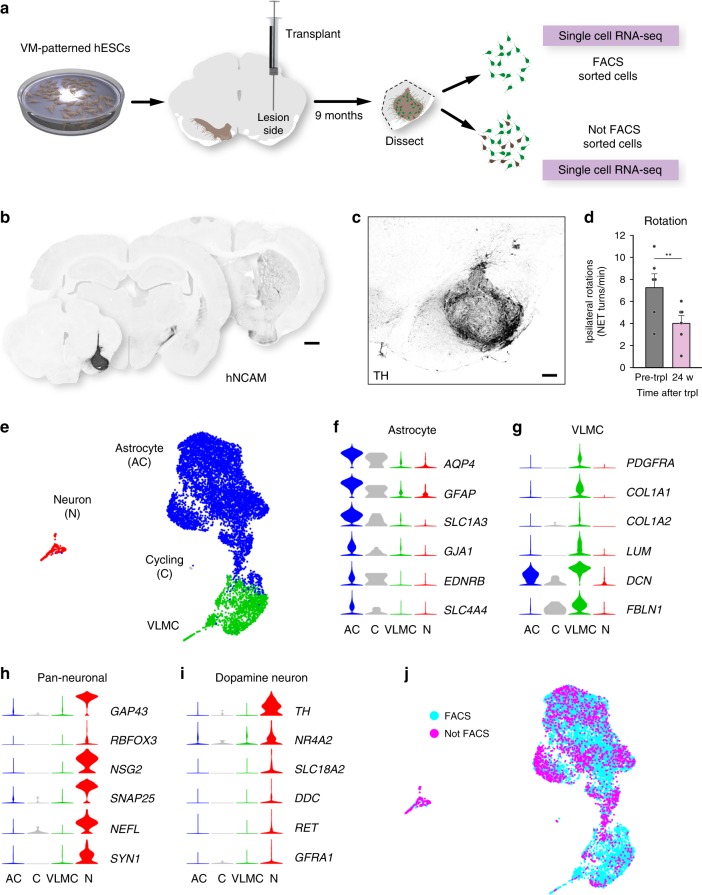
scRNA-seq analysis and histological validation of grafted cells into the midbrain. **a** Schematic overview of experimental design. VM-patterned hESCs grafted to midbrain of 6-OHDA rats and analyzed at 9 months (n = 6). These rats were used as follows: scRNA-seq *n* = 3; histology *n* = 3, functional recovery *n* = 6. **b** Overview of hNCAM fiber outgrowth from hESC-derived intranigral graft showing a neuron-rich graft core and extensive re-innervation of the host striatum. **c** Immunohistochemistry showing TH staining in graft core of a hESC-derived intranigral graft at 9 months post-transplantation. **d** Drug-induced rotation test showing functional recovery in rats that have been transplanted to the midbrain with hESC-derived cells (*n* = 6 rats; mean ± SEM; **p < 0.01; compared to post-lesion; two-tailed paired *t*-test). **e** UMAP embedding showing clustering of 7875 analyzed cells after grafting to the midbrain (grafted rats *n* = 3). **f**–**i** Expression level per cluster for indicated genes. Indicated genes are established markers for astrocytes, VLMCs, neurons, and DA neurons, respectively. All indicated markers are the same as in Fig. 2. **j** UMAP of grafted cells as shown in Fig. 4e. Cells isolated by FACS (blue circles, *n* = 5958) or not by FACS (magenta circles, *n* = 1917) are indicated. Scale bars, 1 mm (**b**); 200 µM (**c**).

The scRNA-seq analysis of grafted cells show that hESC-derived transplanted cells give rise to neurons, astrocytes, and VLMCs, but not to oligodendrocytes. In these experiments, detection of GFP (from the *SYN-GFP* transgene) was used to isolate grafted hESC-derived cells by FACS. To validate and extend the findings, hESCs patterned by the same protocol as initially used (Figs. 1 and 2) were grafted to the midbrain of nude rats (homotopic graft placement) and analyzed by scRNA-seq after 9 months of in vivo maturation (Fig. 4a–d). Importantly, to ensure that the GFP reporter and method of cell isolation did not influence or bias the results, cells in the new experiment were either sequenced directly after dissociation or after FACS isolation (outlined in Fig. 4a). In addition, the 10X Genomics Platform was used to allow for higher throughput. After QC and filtering to exclude rat cells (see Supplementary Fig. 8a–c), a total of 7875 cells were retained for analysis. The resulting UMAP embedding and graph-based clustering showed that, as with the hESC-derived cells grafted to the striatum (Fig. 2), VM-patterned hESCs transplanted to the midbrain gave rise to three main clusters which, based on marker expression, were clearly classified as astrocytes, neurons, and VLMCs (Fig. 4e–i, Supplementary Data 4). A small number of cells expressing astrocyte markers expressed cycling genes and clustered separately (Fig. 4e). Similar results were derived regardless of whether cells had been isolated and sequenced directly or isolated by FACS before sequencing (Fig. 4j).

Next, data from all hESC-derived grafted cells (in total, 8558 cells) was integrated (683 cells grafted to the striatum; Smartseq2 and 7875 cells grafted to the midbrain; 10X Genomics) by using the Seurat data integration strategy^27^. Cells grafted to the striatum (sorted and subjected to Smartseq2 sequencing) and to the midbrain (sorted and unsorted, subjected to 10X Genomics) were all distributed in the main clusters (Supplementary Figs. 9–11; see also enriched markers in Supplementary Data 5). Thus, VM-patterned hESC-derived cells formed the three major cell types (astrocytes, neurons, and VLMCs) regardless of how they were isolated (direct isolation or FACS) and independent of grafting site (striatum or midbrain). In addition, similar to when cells were grafted to the striatum, the midbrain grafts resulted in a low yield of recovered neurons, but in the integrated dataset the total number of analyzed neurons was substantially increased (from 35 to 232 cells) and the neuron cluster expressed *TH* and several other DA neuron markers including *NR4A2*, *SNCA, PBX1*, and *DLK1* (Supplementary Fig. 10f). These markers were broadly distributed within the cluster and separate analysis of neurons alone did not give any indication of separate sub-clustering or marker expression for other neuron types, including serotonergic neurons. However, additional analysis of larger number of recovered neurons will be important to further resolve graft neuron diversity in future studies.

## Discussion

Both fetal and stem cell-derived grafts are highly heterogeneous with respect to cell type composition (reviewed in ref. ^28^). Moreover, all reports of graft composition to date uses histological methods that rely on pre-conceived notions concerning cell types likely to be present in the grafts. This study provides an unbiased assessment of graft composition based on scRNA-seq of fetal and hESC-derived grafts. The number of fetal cells sequenced after transplantation was very low, thus limiting the conclusions that could be derived from these data alone; however, key findings were validated by histological analysis and by analysis of in vitro-cultured cells.

The data confirm that both fetal VM and hESC-derived DA progenitors give rise to neurons and astrocytes after grafting. The results also suggest major differences between the different sources of cells as they demonstrate that oligodendrocytes are only present in fetal cell grafts while grafts of hESC-derived VM-patterned progenitors contain a cell type that was not previously known to be part of neural grafts. These cells resemble normal vascular fibroblasts/VLMCs that were recently identified in the mouse brain using scRNA-seq^16,22^. The graft-derived VLMCs were primarily found localized around blood vessels and intermingled with endogenous vascular cells. scRNA-seq of the cells prior to transplantation showed that markers typical of VLMCs and DA progenitors are expressed by the same cells at the time of transplantation suggesting that the hESC-derived progenitors at this timepoint might have the developmental potential to generate both neural and perivascular cell types. In our analysis of fetal tissue (ranging from gestational week 7.5 to 9) we could not document the presence of VLMCs or their progenitors in the fetal VM itself or in transplants or primary cultures derived thereof, leading us to conclude that VLMCs are most likely a unique component of hPSC-derived VM-patterned DAergic grafts. However, although the developmental origin of these cells remains unknown, previously published scRNA-seq data of human fetal cortical tissue have reported the presence of cells with a transcriptional profile of VLMCs, including COL1A1, COL3A1, and PCOLCE in the early human fetus at age 5–6 weeks pc^29^, and it can therefore not be ruled out that VLMCs may be a component of early neural development. It will clearly be intriguing in future studies to establish the origin of VLMCs, both under normal development and in cultures of differentiating hESCs.

Efforts to use stem cell-derived DA neurons in clinical applications have progressed immensely and they are now on the verge of entering clinical trials^3^. In addition to providing an unbiased and comprehensive characterization of the cells in the graft, the scRNA-seq analysis also resulted in several findings with significant implications for transplanting hPSC-derived VM progenitors in patients. First, scRNA-seq of cells after grafting demonstrated that only very few hESC-derived cells belonging to the astrocyte cluster remained proliferative. Second, serotonergic neurons that could potentially cause dyskinesias were not evident in hESC-derived grafts^30,31^. Thus, the transcriptional analysis presented here provides support to the conclusion that these cells are safe to use in cell therapy. The discoveries presented in this study enables future focused studies to investigate the contribution of each individual cell type to graft integration and function. Further studies using grafts of controlled compositions are needed to better understand the role of astrocytes, oligodendrocytes, and VLMCs in graft vascularization, survival, maturation, and integration of DA neurons.

## Methods

### Animals

Athymic nude female rats (180 g) were purchased from Harlan laboratories (Hsd:RH-Foxn1_rnu_) and housed in individual ventilated cages under a 12:12 h dark–light cycle with ad libitum access to sterilized food and water. All procedures on research animals were in accordance with the European Union directive (2010/63/EU) and approved by the local ethical committee at Lund University as well as the Swedish Department for Agriculture (Jordbruksverket). For all surgical procedures, rats (minimum 225 g/16–18 weeks) were anesthetized via i.p. injections using a 20:1 mixture of fentanyl-dormitor (Apoteksbolaget). The animals were rendered hemiparkinsonian via unilateral injection of 4 µL of the neurotoxin 6-hydroydopamine at a concentration of 3.5 µg/µL (calculated from free-base HCl) aimed at the following stereotaxic coordinates (in mm): anterior: −4.4, medial: −1.2, and dorsal: −7.8 with the incisor bar set at 2.4. Injections were performed as described^32^ at an injection speed of 1 µL/min with an additional 3 min allowed for diffusion before careful retraction of the needle. Drug-induced rotations were assessed 4 weeks post-lesion (d-amphetamine, 2.5 mg/kg, i.p., Apoteksbolaget, Sweden) for 90 min in automated rotometer bowls (Omnitech Electronic Inc., USA). Rotational data were expressed as net turns per minute. The cylinder test was also used by placing animals in a glass cylinder (diameter 30 cm) for 5 min while video recording. We counted the total number of touches the animals performed over the 300 s interval with the paw ipsilateral and contralateral to the lesion/graft, respectively. Paw use preference was expressed as bias score. After matching rats into groups according to their behavioral performance each rat received a single-cell suspension of the respective hESC or human VM tissue. For intrastriatal grafts of hESCs-derived VM-patterned cells, we injected a total of 300,000 cells in 4 µL over four 1 µL deposits (75,000 cells per deposit) at the following coordinates relative to bregma (in mm): anterior: +1.2, medial: −2.6, dorsal: −4.5 and −4.0 and anterior: +0.5, medial: −3.0.

For intranigral grafts of hESC-derived VM progenitors, we injected a total of 150,000 cells over two deposits (75,000 cells per deposit) to the midbrain at the following coordinates AP: −5.2; ML −2.3; DV −6.0/−6.5; TB flat head. At each deposit the injection-cannula was left in place for an additional 2 min before careful retraction to allow for settling of the tissue. The maturation of the graft was monitored using d-amphetamine-induced rotations as described above. On the day before perfusion a second cylinder test was performed. After behavioral data had been collated, the rats were transcardially perfused using 50 mL physiological saline solution (8.9% saline) over 1 min followed by 250 mL 4% PFA (pH 7.4) solution over 5 min. For hESC-derived DA progenitors eight animals were transplanted in the intrastriatal grafting experiments. Out of eight, seven rats had surviving grafts and were used for analysis (two for sequencing and five for histology). For the intranigral transplants, nine rats were transplanted: one died after surgery but the remaining eight all had surviving grafts: two were used to ensure good graft survival and maturation after 6 months and the remaining six were kept until the 9-month endpoint. At this time three were used for sequencing and three for histology.

### Human fetal tissue preparation

Human fetal tissue from legally terminated embryos was collected in accordance with existing guidelines with approval of the Swedish National Board of Health and Welfare and informed consent from women seeking elective abortions. To determine the gestational age of the embryos, the crown-to-rump length was measured and the embryo were staged according to week post-conception.

Fetal VM tissue (7,5 weeks) was prepared for transplantation according to the same standard operating procedures (SoPs) as were approved for the clinical TRANSEURO trial. Fetal VM was dissected, cut into smaller pieces, and incubated in TrypLE Select/Dornase alpha (DA, 20 U/mL) for 20 min at 37 °C for further dissociation. To achieve a crude cell suspension the tissue was then triturated first with a large tip (1000 µL) and then with a small tip (100 µL) in HBSS/Tirilazad Mesylate (TM)/DA. The cells were finally resuspended in 24 µL HBSS/TM/DA which is the same medium used for transplanting the hESC-derived progenitors. One VM was transplanted to three rats. For the experiment used for sequencing, the two rats used to isolate grafts had reduced rotational scores (one from 6.29 to 0.14 and the other from 4.35 to −0.91) indicating functionality of the grafts.

Fetal VM tissue from two embryos (7.5 and 9 weeks) were dissected and prepared using same protocol and subsequently plated at a density of 27,800 cells per cm^2^ in 24-well plates (Nunc). Prior to plating, the wells were coated overnight with a combination of polyornithine (1.5 mg/mL), fibronectin (0.5 mg/mL), and Laminin (1 mg/mL). Cells were kept in culture with neuronal media (Neurobasal) supplemented with B27 without vitamin A (20 mL/L), l-glutamine (2 mM), penicillin/streptomycin (0.2%), BDNF (20 ng/mL), GDNF (10 ng/mL), ascorbic acid (0.2 mM) and maintained at 37 °C in 5% CO_2_.

### hESC differentiation

hESCs (RC17: hPSCreg RCe021-A; H9: hPSCreg WAe009-A; HS980a: hPSCreg KIe033-A) and iPSCs (Miltenyi) were differentiated into VM-patterned progenitors using both our research-grade differentiation protocol (H9)^7,19^ and our GMP-grade protocol (RC17 and HS980a)^4^ and transplanted after 16 days of differentiation. Only cell preparations that could be approved according to a pre-determined quality range^4^ were used for grafting. For expression of nuclear expression of GFP, RC17 hESCs (passage 25) were transduced with a lentiviral construct expression a histone H2B-GFP fusion protein under control of the human synapsin promoter (Addgene plasmid # 30456). The cells were transduced at a multiplicity of infection (MOI) of 6 while cultured on Laminin-521 in iPS-Brew (Miltenyi Biotec). For terminal differentiation, the VM-patterned progenitors were replated on day 16 as described^4^ and kept in culture until day 42–52 of differentiation.

To further assess the presence of VLMCs after in vitro differentiation we adopted two additional recently published/patented protocols that are intended for clinical use^12,26^ (see also https://patents.justia.com/patent/20180094242). For VM-patterned organoid differentiation RC17 hESCs were differentiated into self-organized organoids as described^33^, in presence of CHIR (CHIR99021) and SHH (SHH-C24II) for midbrain patterning^8^.

### Tissue dissociation and FACS sorting

Grafted human fetal VM and grafted VM-patterned hESCs cells were dissected from rat striatum (*n* = 2 for fetal VM intrastriatal grafts, *n* = 2 for hESC intrastriatal grafts, and *n* = 3 for intranigral grafts) as described^34^ and dissociated into a single-cell suspension using the papain kit (Worthington) following the manufacturer’s instructions. After dissociation, the cells were FACS sorted based on size (fetal tissue) or GFP expression (VM-patterned hESCs) using BD FACSAria III Cell Sorter (Supplementary Fig. 1g, h). The single-cell suspension of human fetal VM and VM-patterned hESCs before grafting were FACS sorted based on size. Tissue from individual animals were processed and sequenced separately.

### Library preparation and sequencing

The cDNA libraries from the sorted cells were generated using the Smartseq2 protocol^35^. cDNA libraries were tagmented using a home-made Tn5 enzyme^36^ and Nextera dual indexes (i5 + i7). The quality of cDNA and tagmented cDNA was checked on a High-Sensitivity DNA chip (Agilent Bioanalyzer). Illumina HiSeq 2000 was used for sequencing, giving 43 base pair reads after de-multiplexing.

For 10× samples, single-cell suspensions were loaded onto 10X Genomics Single Cell 3′ Chips along with the reverse transcription (RT) mastermix per the manufacturer’s protocol for the Chromium Single Cell 3′ Library to generate single-cell gel beads in emulsion (GEMs). cDNA amplification was done as per the guidelines from 10X Genomics. Sequencing libraries were generated with unique sample indices (SI) for each sample with the following specifications Read1 28 cycles, Read2 98 cycles, Index1 8 cycles. Libraries for samples were multiplexed and sequenced on an Illumina NextSeq 500 machine using a 150-cycle NextSeq 500/550 High Output Reagent Kit v2. Sample Rat39_3a from Not FACS sorting was split into half (Rat39_3a and Rat39_3c) prior to cDNA amplification and was sequenced on separate lanes. Counts matrices obtained from these two samples were merged (rat39-3ac) during the downstream analysis.

### Read alignment and quality control

Reads for Smartseq2 data were aligned to the human genome (hg19) merged with *GFP* and TVA sequences using STAR v2.3.0 (ref. ^37^) and filtered for uniquely mapping reads. Gene expression was calculated as reads per kilobase gene model and million mappable reads (RPKMs) for each transcript in Ensembl release 69 using rpkmforgenes^38^. To exclude the contaminated rat cells, kallisto software was used for mapping of reads to the rat genome. The low-quality libraries were filtered out based on following parameters: <5% uniquely mapping reads, >25% fraction mismatches, <10% exon mapping reads, >20% 3′ mapping, <3% or >30% of all genes detected, <10,000 normalization reads, >10% reads mapped to rat genome (Supplementary Fig. 1i). From 2206 sequenced cells 1406 passed the quality control.

For 10X Genomics samples, raw base calls were demultiplexed and converted to sample-specific fastq files using cellranger mkfastq program. Raw reads were processed using cellranger count version 3 pipeline using default parameters. Precisely, this pipeline uses STAR to map cDNA reads to the transcriptome (GRCh38). Only exonic reads that uniquely mapped to transcriptome were used for the downstream analysis. Aligned reads were filtered for valid barcodes and UMI and observed cell barcodes were retained if they were 1-Hamming-distance away from entering into the whitelist of barcodes. Non-homopolymer UMIs with quality score greater than 10 were retained. Distribution of UMIs in each unique cell barcodes was examined and cell barcodes with UMI counts that fell within the 99th percentile of the defined range of estimated cell count were selected. Cells were excluded if more than 10% reads aligned to Rat genome (Rnor6) (Supplementary Fig 8c). Low-quality cells were filtered out based on: (a) fraction of UMIs mapping to mitochondria larger than 0.15 and (b) log10 detected genes per cell (nGene) below mean minus two standard deviations for each sample separately (Supplementary Fig 8b). A total of 7875 cells were used for downstream analysis.

### Smartseq2 cell population definition

Seurat package^39^ version 2.2.0 was used to normalize the data by gene detection and to extract the variable genes which were used for downstream analysis (x.low.cutoff = 1, x.high.cutoff = 10, y.cutoff = 1). Only statistically significant principal components were included (cells after grafting-PC: 1–12, cells before grafting-PC: 1:6, 8, 10:12). Clusters have been defined with Seurat function FindClusters using resolution 0.6. This gave five clusters for the cells before grafting (Supplementary Fig. 2a) and seven clusters for the cells after grafting (Supplementary Fig. 4a). After analysis of differentially expressed genes between the clusters, clusters with few differentially expressed genes between them and close proximity on the t-SNE were manually defined as a single cluster, which gave four clusters for cells before grafting and four clusters for cells after grafting (Figs. 1e and 2a). The clustering results were visualized with the t-SNE plot (Rtsne: T-Distributed Stochastic Neighbor Embedding using a Barnes-Hut implementation, https://github.com/jkrijthe/Rtsne). To extract the cluster-specific genes we used the SAMseq software package^13^. Differentially expressed genes for each cluster are in Supplementary Data 1 and 2. Genes were considered significantly differentially expressed if *q*-value < 0.01. To analyze the effect of the cell cycle on the cluster separation the CellCycleScoring was regressed out during Seurat data scaling analysis (Supplementary Fig. 2j, k). Only statistically significant principal components were included (PC: 1, 2, 4, 5, 7, 9, 10, 12, 13) for the t-SNE clustering. FindClusters Seurat function was used to define clusters (resolution 0.6).

### Integrated analysis

All clustering and analysis was performed with Seurat v3.0.0 (ref. ^27^) (Fig. 4e, Supplementary Figs. 8d and 9a). Cells from each rat graft was merged into individual datasets as well as grafted cells originating from hESCs in the SmartSeq2 dataset. Each dataset was log normalized and scaled with regression of number of features. The four datasets were integrated using the strategy described in Stuart et. al. using 30 principal components and 2000 anchor features. Dimensionality reduction was performed with UMAP^40^ using 30 principal components. Cells were split into four clusters using Seurat default SNN clustering with resolution 0.1. Differential expression was performed with the Wilcoxon rank-sum test (Supplementary Data 4 and 5). Genes were considered significantly differentially expressed if *p*_adj_ value < 0.05.

### Pathway analysis

The gene clusters after transplantation (genes from Supplementary Data 2) were analyzed for biological functions using the Ingenuity Pathway Analysis program (QIAGEN Inc., https://www.qiagenbioinformatics.com/products/ingenuity-pathway-analysis). Only functional categories related to System Development functions were selected (Supplementary Data 3) and ten most significant System Development functions of each cluster were plotted in Supplementary Fig. 6a.

### Comparison with other datasets

Gene expression profiles per cluster from the Mouse brain atlas^15^ was downloaded from their website (http://mousebrain.org). A marker gene set consisting of top 100 up-regulated gene per cluster among the cells after grafting (for either Smartseq2 data alone or integrated with 10x data), combined with marker genes for all the 256 cell types in the atlas was used in the comparison (Supplementary Figs. 6b, c and 11). The 256 atlas cell types were grouped into main clusters at Taxonomy rank 4 (39 groups) and mean expression per group was calculated using the marker gene set. These were compared to the mean expression in our clusters using spearman correlation.

### Cell cycle scores

G2M and S phase scores were calculated using the function CellCycleScoring in the Seurat package. As described^41^ the cells were classified as cycling if either of the scores were larger than 1. If scores were less than 1 they were defined as non-cycling.

### Immunohistochemistry

After perfusion, brains were post-fixed for 24 h in 4% PFA and then cryopreserved in a 30% sucrose solution before being sectioned coronally on a freezing sledge microtome at a thickness 35 µm in series of 1:8 or 1:12.

Immunohistochemistry was performed on free-floating sections and all washing steps were done with 0.1 M phosphate-buffered saline with potassium (KPBS, pH = 7.4). The sections were washed three times and then incubated in Tris-EDTA pH8 for 30 min at 80 °C for antigen retrieval. After washing an additional three times, the sections were incubated in blocking solution (KPBS + 5% serum of species the secondary AB was raised in +0.25% Triton X-100) for 1 h, before adding the primary antibody solution. The primary antibodies used were: mouse anti-HuNu (1:200, Merck Millipore, cat. no. MAB1281), mouse anti-hNCAM (1:1000, Santa Cruz Biotechnology, cat. no. sc-106), rabbit anti-TH (1:1000, Merck Millipore, cat. no. AB152), rabbit anti-NeuN (1:500, Merck Millipore, cat. no. ABN78), sheep anti-hCOL1A1 (1:200, R&D, cat.no. AF6220), rabbit anti-COL1A1 (1:200, Abcam, cat. no. ab34710), rabbit anti-hPDGFRα (1:300, Cell Signaling, cat. no. 5241), rabbit anti-Olig2 (1:500, Neuromics, cat. no. RA25081), and chicken anti-GFP (1:1000, Abcam, cat. no. ab13970). After incubation with primary antibodies overnight at room temperature (RT), the sections were washed twice and incubated in blocking solution for 30–45 min. For fluorescent immunolabeling, the sections were then incubated with fluorophore-conjugated secondary antibodies (1:200, Jackson ImmunoResearch Laboratories) for 2 h at RT, washed three times and then mounted on gelatin-coated slides and coverslipped with PVA-DABCO containing DAPI (1:1000). For di-amino-benzidine (DAB) stainings, the sections were incubated with secondary biotinylated-horse antibodies (1:200, Vector Laboratories) for 1 h at RT, washed three times, and then incubated with avidin-biotin complex (ABC) for 1 h at RT for amplification. Peroxidase driven precipitation of DAB was used to detect the primary antibody. In this step, sections were incubated in 0.05% DAB for 1–2 min before addition of 0.01% H_2_O_2_ for 1–2 min. After development of the DAB staining, the sections were mounted on gelatin-coated slides and then dehydrated in an ascending series of alcohols, cleared in xylene, and coverslipped with DPX mountant.

The hESC graft in Figs. 1b and 3b–f and Supplementary Fig. 1d–f has previously been analyzed for a different purpose and published^4,11,25^. The micrographs presented here are derived uniquely for this study.

### Immunocytochemistry

Terminally differentiated cell cultures (days 42–52) were fixed in 4% PFA for 15 min and then washed three times with PBS. For immunocytochemistry, the cells were blocked for 1–3 h in PBS + 5% donkey serum + 0.1% Triton X-100 before adding the primary antibodies solution. The primary antibodies included: rabbit anti-TH (1:1000, Merck Millipore, cat. no. AB152), sheep anti-hCOL1A1 (1:200, R&D, cat.no. AF6220), and rabbit anti-hPDGFRα (1:300, Cell Signaling, cat. no. 5241). After incubation with primary antibodies overnight at 4 °C, the cells were washed three times before adding fluorophore-conjugated secondary antibodies (1:200, Jackson ImmunoResearch Laboratories) and DAPI (1:500). The cultures were incubated with secondary antibodies for 2 h and finally washed three times.

### Microscopy and images

Images were captured using either a flatbed scanner Epson Perfection V850 PRO, a Leica DMI6000B widefield microscope or a Leica TCS SP8 laser scanning confocal microscope. The image acquisition software was Leica LAS X and images were processed using Volocity 6.5.1 (Quorum Technologies) and Adobe Photoshop. Any adjustments were applied equally across the entire image, and without the loss of any information. The following images were digitally stitched from multiple images: Figs. 1b, 2h, 3a–f, 4b, c, Supplementary Figs. 1k, 5b and  7d, e, g–j. Figures 1a and 4a were created by the authors. Each image represents a typical result obtained from analysis of minimum 2–3 graft sections per animal.

### Reporting summary

Further information on research design is available in the Nature Research Reporting Summary linked to this article.

## Supplementary information

Reporting Summary

Supplementary Information

Description of Additional Supplementary Files

Supplementary Data 1

Supplementary Data 2

Supplementary Data 3

Supplementary Data 4

Supplementary Data 5

## Data Availability

The authors declare that all data supporting the findings of this study are available within the article and its supplementary information files or from the corresponding author upon reasonable request. Raw data have been deposited in the GEO databse under accession codes: GSE118412 and GSE132758. An application for analyzing single cell data is available at http://perlmannlab.org. The source data underlying Figs. 1c, 1d, 4d and Supplementary Fig. 5c are provided as a Source Data file.
