# Author Correction: Single cell transcriptomics identifies stem cell-derived graft composition in a model of Parkinson’s disease

**DOI:** 10.1038/s41467-020-17421-z

**Published:** 2020-07-15

**Authors:** Katarína Tiklová, Sara Nolbrant, Alessandro Fiorenzano, Åsa K. Björklund, Yogita Sharma, Andreas Heuer, Linda Gillberg, Deirdre B. Hoban, Tiago Cardoso, Andrew F. Adler, Marcella Birtele, Hilda Lundén-Miguel, Nikolaos Volakakis, Agnete Kirkeby, Thomas Perlmann, Malin Parmar

**Affiliations:** 1 Ludwig Institute for Cancer Research, Box 240, SE-171 77 Stockholm, Sweden; 2 Department of Cell and Molecular Biology, Karolinska Institutet, SE-171 77 Stockholm, Sweden; 3 Developmental and Regenerative Neurobiology, Wallenberg Neuroscience Center, and Lund Stem Cell Centre, Department of Experimental Medical Science, Lund University, 22184 Lund, Sweden; 4 Present Address: Department of Cell and Molecular Biology, National Bioinformatics Infrastructure Sweden, Science for Life Laboratory, Uppsala University, Husargatan 3, SE-752 37 Uppsala, Sweden; 5 Novo Nordisk Foundation Center for Stem Cell Biology (DanStem), University of Copenhagen, 2200 Copenhagen, Denmark

**Keywords:** Embryonic stem cells, Embryonic stem cells, Regeneration

Correction to: *Nature Communications* 10.1038/s41467-020-16225-5, published online 15 May 2020.

The original version of this Article contained an error in the Acknowledgments, which incorrectly read “The research leading to these results has received funding from the New York Stem Cell Foundation (to M.P.), the European Research Council (ERC Grant Agreement no. 30971, to M.P.), the Swedish Research Council [grant agreements 521-2012-5624 (to M.P.), 2016-02506 (to T.P.) and 70862601/Bagadilico)], Swedish Parkinson Foundation (Parkinsonfonden, to M.P.), Swedish Brain Foundation (Hjärnfonden, to M.P.), the Strategic Research Area at Lund University Multipark (Multidisciplinary research in Parkinson’s disease), StratRegen grant Karolinska Institutet (to T.P.); Knut and Alice Wallenberg Foundation (to T.P.), Söderbergs Stiftelse (to T.P.) and Svenska Sällskapet för Medicinsk Forskning (to K.T.). M.P. is a New York Stem Cell Foundation—Robertson Investigator.’

The correct version states ‘ERC Grant Agreement no. 771427, to M.P.’ in place of ‘ERC Grant Agreement no. 30971, to M.P.’

This has been corrected in both the PDF and HTML versions of the Article.

