# Supplementary material for: Single cell transcriptomics identifies stem cell-derived graft composition in a model of Parkinson’s disease
Source: Nat Commun. 2020 May 15;11:2434. doi: 10.1038/s41467-020-16225-5 (PMC7229159; doi:10.1038/s41467-020-16225-5)
Supplement: Supplementary file 1 — Reporting Summary [file 41467_2020_16225_MOESM1_ESM.pdf]

nature research

Corresponding author(s): Malin Parmar, Thomas Perlmann

Reporting Summary

Nature Research wishes to improve the reproducibility of the work that we publish. This form provides structure for consistency and transparency in reporting. For further information on Nature Research policies, see Authors & Referees and the Editorial Policy Checklist.

Statistical parameters

When statistical analyses are reported, confirm that the following items are present in the relevant location (e.g. figure legend, table legend, main text, or Methods section).

n/a	Confirmed	
☐	☒	The exact sample size (*n*) for each experimental group/condition, given as a discrete number and unit of measurement
☐	☒	An indication of whether measurements were taken from distinct samples or whether the same sample was measured repeatedly
☐	☒	The statistical test(s) used AND whether they are one- or two-sided<br>*Only common tests should be described solely by name; describe more complex techniques in the Methods section.*
☒	☐	A description of all covariates tested
☒	☐	A description of any assumptions or corrections, such as tests of normality and adjustment for multiple comparisons
☒	☐	A full description of the statistics including central tendency (e.g. means) or other basic estimates (e.g. regression coefficient) AND variation (e.g. standard deviation) or associated estimates of uncertainty (e.g. confidence intervals)
☒	☐	For null hypothesis testing, the test statistic (e.g. *F*, *t*, *r*) with confidence intervals, effect sizes, degrees of freedom and *P* value noted<br>*Give P values as exact values whenever suitable.*
☒	☐	For Bayesian analysis, information on the choice of priors and Markov chain Monte Carlo settings
☒	☐	For hierarchical and complex designs, identification of the appropriate level for tests and full reporting of outcomes
☒	☐	Estimates of effect sizes (e.g. Cohen's *d*, Pearson's *r*), indicating how they were calculated
☐	☒	Clearly defined error bars<br>*State explicitly what error bars represent (e.g. SD, SE, CI)*

Our web collection on statistics for biologists may be useful.

Software and code

Policy information about availability of computer code

Data collection	Star v2.3.028, Kallisto v0.43.0, RSeQC v2.3.9, rpkmforgenes.py, cellranger v3.0.0
Data analysis	All code for producing main figures of the paper is available at: https://github.com/asabjorklund/DA_grafting_analysis<br>Softwares used:<br>SmartSeq2 data: Seurat v2.3.4, samr v2.0, R v3.4.1, Ingenuity Pathway Analysis (Qiagen)<br>10Xdata and integrated data (SmartSeq2 & 10X): cellranger v3.0.0, Seurat v3.0.0, R v3.5.1

For manuscripts utilizing custom algorithms or software that are central to the research but not yet described in published literature, software must be made available to editors/reviewers upon request. We strongly encourage code deposition in a community repository (e.g. GitHub). See the Nature Research guidelines for submitting code & software for further information.

Data

Policy information about availability of data

All manuscripts must include a data availability statement. This statement should provide the following information, where applicable:

- Accession codes, unique identifiers, or web links for publicly available datasets
- A list of figures that have associated raw data
- A description of any restrictions on data availability

To review GEO accession
GSE118412: https://www.ncbi.nlm.nih.gov/geo/query/acc.cgi?acc=GSE118412
GSE132758 : https://www.ncbi.nlm.nih.gov/geo/query/acc.cgi?acc=GSE132758

Field-specific reporting

Please select the best fit for your research. If you are not sure, read the appropriate sections before making your selection.

[x] Life sciences [ ] Behavioural & social sciences [ ] Ecological, evolutionary & environmental sciences

For a reference copy of the document with all sections, see nature.com/authors/policies/ReportingSummary-flat.pdf

Life sciences study design

All studies must disclose on these points even when the disclosure is negative.

Sample size	In this study, we aimed to sequence as many single cells before grafting and after grafting. We achieved 2206 cells for Smartseq2 data and 8519 for 10X data. No statistical methods were used to predetermine sample size.
Data exclusions	Animals with no grafts as determined by histology at the end of the experiment were excluded from analysis. For the single cells we have used the following cell exclusion criteria:<br>Smartseg2 data:< 5% uniquely mapping reads, > 25% fraction mismatches, < 10% exon mapping reads, >20% 3'mapping, < 3% or > 30% of all genes detected, < 10000 normalization reads, > 20% reads mapped to rat genome. From 2206 sequenced cells 800 cells were excluded.<br>10X data: Cells were excluded if more than 10% reads aligned to Rat genome (Rnor6). Low quality cells were filtered out based on: a) fraction of UMIs mapping to mitochondria larger than 0.15 and b) log10 detected genes per cell (nGene) below mean minus two standard deviations for each sample separately. A total of 8519 cells were used for downstream analysis.
Replication	All major findings has been validated in animlas from at least 3 separate experiments, using 4 different cell lines.
Randomization	N/A
Blinding	Behavioral testing of rats performed by blinded investigator

Reporting for specific materials, systems and methods

Materials & experimental systems

n/a	Involved in the study
[ ]	[x] Unique biological materials
[ ]	[x] Antibodies
[ ]	[x] Eukaryotic cell lines
[x]	[ ] Palaeontology
[ ]	[x] Animals and other organisms
[x]	[ ] Human research participants

Methods

n/a	Involved in the study
[x]	[ ] ChIP-seq
[ ]	[x] Flow cytometry
[x]	[ ] MRI-based neuroimaging

Unique biological materials

Policy information about availability of materials

Obtaining unique materials	The study includes human fetal tissue. It was collected from a legally terminated embryo collected in accordance with existing guidelines with approval of the Swedish National Board of Health and Welfare and informed consent from women seeking elective abortions.

Antibodies

Antibodies used	Please see Supplementary Table 1.
Validation	Please see manufacturer information.

Eukaryotic cell lines

Policy information about cell lines

Cell line source(s)	RC17: hPSCreg RCe021-A obtained from Roslin Cells<br>iPS-line: BJ-hFFs using mRNA reprogramming from Miltenyi<br>H9: hPSCreg WAe009-A obtained from WiCell<br>HS980a: hPSCreg KIe033-A, obtained from Outi Hovatta, Karolinska Institute
Authentication	N/A
Mycoplasma contamination	all cell lines tested negative for mycoplasma comtamination
Commonly misidentified lines (See ICLAC register)	N/A

Animals and other organisms

Policy information about studies involving animals; ARRIVE guidelines recommended for reporting animal research

Laboratory animals	Athymic nude female rats. Minimum 16 weeks at start of experiment (180 g). Purchased from Harlan Laborotories
Wild animals	N/A
Field-collected samples	N/A

Flow Cytometry

Plots

Confirm that:

- [x] The axis labels state the marker and fluorochrome used (e.g. CD4-FITC).
- [x] The axis scales are clearly visible. Include numbers along axes only for bottom left plot of group (a 'group' is an analysis of identical markers).
- [x] All plots are contour plots with outliers or pseudocolor plots.
- [ ] A numerical value for number of cells or percentage (with statistics) is provided.

Methodology

Sample preparation	Grafted human fetal ventral midbrain and grafted VM-patterned hESCs cells were dissected from rat striatum or substantia nigra and dissociated into a single cell suspension using the papain kit (Worthington).
Instrument	BD FACSAria III Cell Sorter
Software	FlowJo v10.4.2
Cell population abundance	Purity of samples is assessed from the single cell transcriptomes .
Gating strategy	Cells before grafting and grafted human fetal ventral midbrain cells were sorted based on the cell size using forward scatter area/side scatter area (FSC-A/ SSC-A), side scatter width/side scatter area (SSC-W/ SSC-A) and forward scatter area/forward scatter width (FSC-A/ FSC-W). Grafted human fetal ventral midbrain cell suspension was contaminated with the rat cells due to the dissection procedure. The rat cells were later eliminated based on the rat transcriptome.

Grafted VM-patterned hESCs cells were sorted using forward scatter area/side scatter area (FSC-A/ SSC-A), side scatter width/ side scatter area (SSC-W/ SSC-A), forward scatter area/forward scatter width (FSC-A/ FSC-W) followed by detection of fluorescent events FITC-A/PE-A. GFP was excited with a 488 nm laser. Non-fluorescent region (cortex) of the same animal was used as a negative control to set the threshold for fluorescent event selection.
A figure describing the gating strategy is shown in Supplementary Figure 1g, h.

☒ Tick this box to confirm that a figure exemplifying the gating strategy is provided in the Supplementary Information.
