# Supplementary material for: Single cell transcriptomics identifies stem cell-derived graft composition in a model of Parkinson’s disease
Source: Nat Commun. 2020 May 15;11:2434. doi: 10.1038/s41467-020-16225-5 (PMC7229159; doi:10.1038/s41467-020-16225-5)
Supplement: Supplementary file 2 — Supplementary Information [file 41467_2020_16225_MOESM2_ESM.pdf]

Supplementary Information

Tiklová et.al., **Single Cell Transcriptomics Identifies Stem Cell-Derived Graft Composition in a Model of Parkinson's Disease**

Supplementary Figures 1-11

Supplementary Table 1

Supplementary References

Supplementary Figure 1

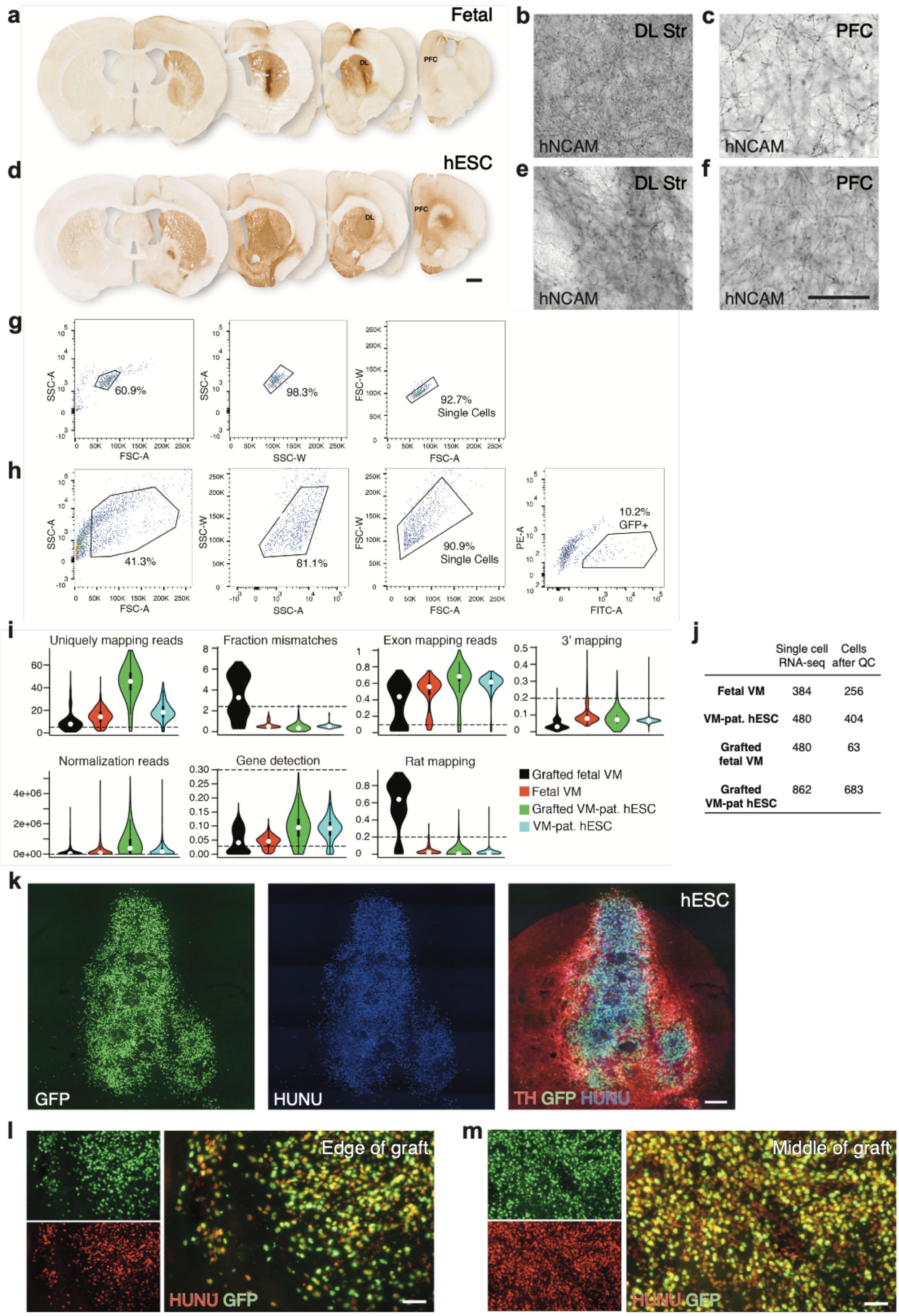

	Single cell RNA-seq	Cells after QC
Fetal VM	384	256
VM-pat. hESC	480	404
Grafted fetal VM	480	63
Grafted VM-pat hESC	862	683

Supplementary Figure 1. Isolation of cells by FACS, quality control of sequenced cells and histological validation of graft

a-f, Overview of the pattern of hNCAM axonal outgrowth derived from fetal- (a-c) and hESC-derived (d-f) intrastriatal grafts 6 months post-transplantation. DL Str – dorsolateral striatum, PFC – prefrontal cortex.

g, FACS strategy for sorting of the single cells based on the cell size. FSC-A: Forward Scatter area; FSC-W: Forward Scatter width; SSC-A: Side Scatter area; SSC-W: Side scatter width; FITC-A: Area of fluorescent dye fluorescein; PE-A: Area of fluorescent dye phycoerythrin.

h, FACS strategy for sorting of the single cells based on the GFP expression. Abbreviations as in g.

i, Filtering of low quality cells based on specified parameters.

j, Table indicating the number of single cells used for scRNA-seq and single cells which passed the QC and were included for further analysis.

k-m, Immunohistochemistry showing GFP (from the reporter) and HUNU staining in the grafts 6 months after transplantation, at the graft edge (e) and in the graft core (f).

Scale bars, 1 mm (a, d), 200 µM (b, c, e, f), 250 µM (k) and 50 µM (l and m).

Supplementary Figure 2

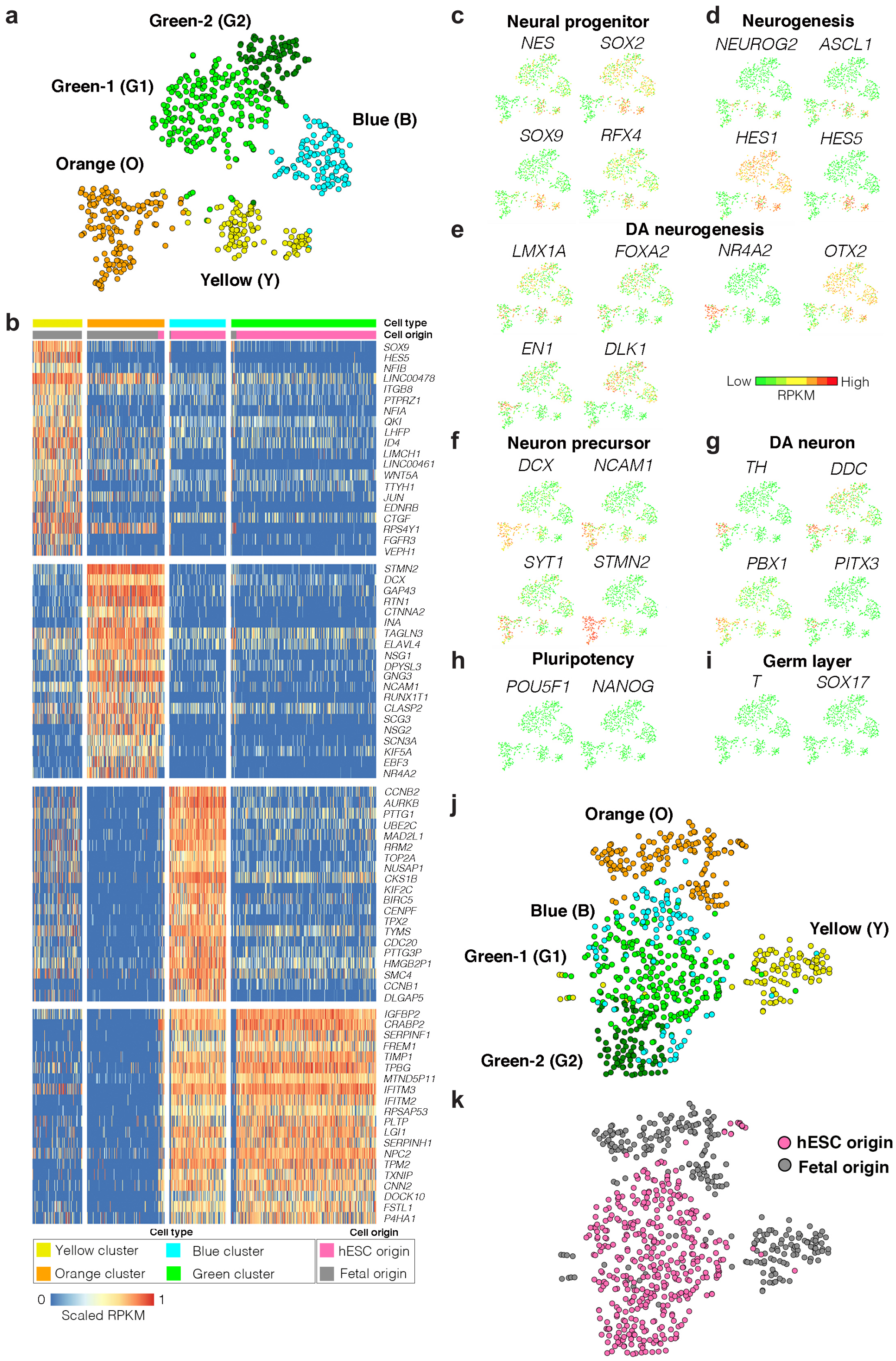

Supplementary Figure 2. Analysis of scRNA-seq data from cells before grafting
a, t-SNE showing clustering of 660 analyzed cells before grafting (404 hESCs origin and 256 fetal origin). Five clusters were defined with Seurat FindClusters. After analysis of differentially expressed genes and visualization by t-SNE, cells were manually defined as 4 clusters as indicated in Fig. 1e and described in Methods.
b, Heatmap visualizing expression of top-enriched genes in clusters of the cells before grafting.
c-i, Expression of markers visualized on the t-SNE plot. The colors indicate the RPKM values.
j, t-SNE showing clustering of 660 cells before grafting after the regression of cell cycle effect. Cells are colored by clusters from a).
k, Same t-SNE as in j) but with origin of cells marked with pink circles (hESC) or grey circles (fetal) as indicated.

Supplementary Figure 3

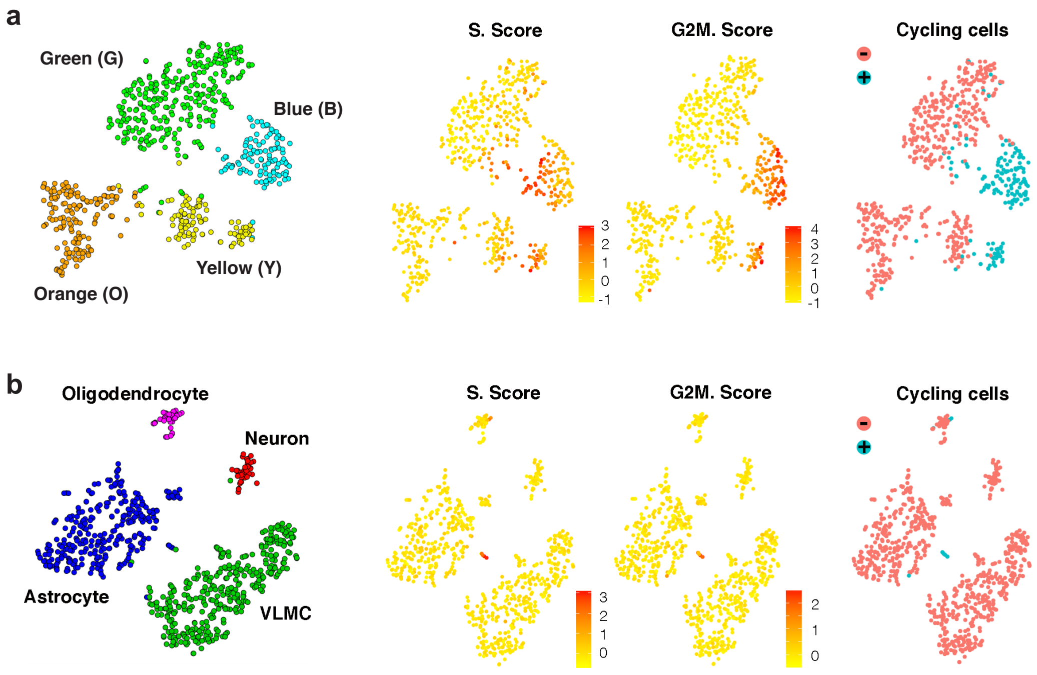

Supplementary Figure 3. Cell cycle scores in cells before and after grafting
a, t-SNE plots showing clusters of cell types before grafting (n=660), cell cycle scores of the analyzed cells (S.Score and G2M.Score) and cycling cells (blue circles). G2M and S cell cycle scores were calculated using the function CellCycleScoring in the Seurat package.
b, t-SNE plots showing clusters of cell types after grafting (n=746), cell cycle scores of the analyzed cells (S.Score and G2M.Score) and cycling cells (blue circles).

Supplementary Figure 4

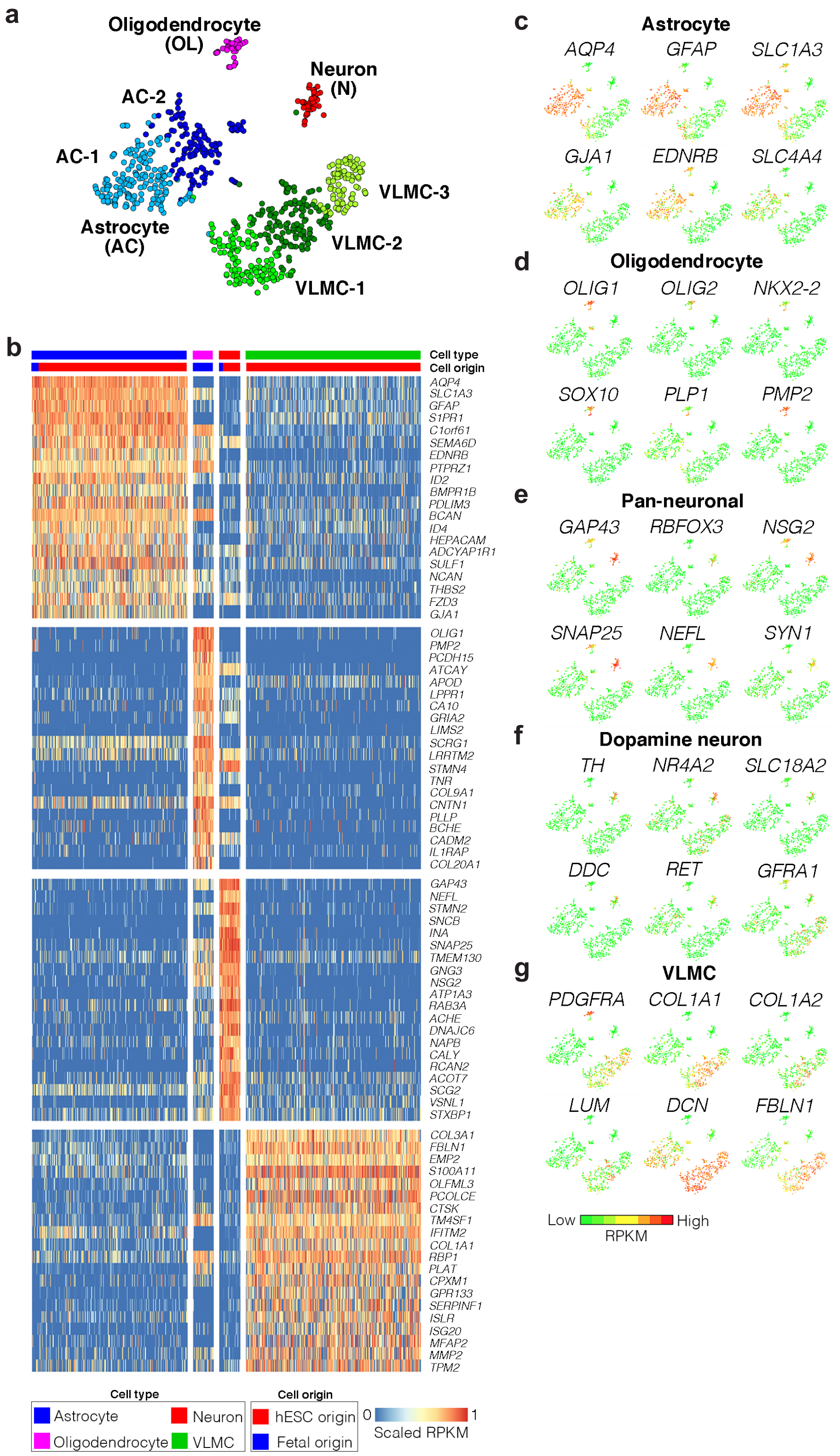

Supplementary Figure 4. **Analysis of scRNA-seq data from grafted cells into the striatum**

a, t-SNE showing clustering of 746 analyzed cells after grafting (683 cells of hESCs origin, grafted rats n=2 and 63 cells of fetal origin, grafted rats n=2). Seven clusters were defined

with Seurat FindClusters. After analysis of differentially expressed genes and visualization by t-SNE, cells were manually defined as 4 clusters as indicated in Fig. 2a and described in Methods. The clusters were assigned to different cell types as described for Fig. 2.
b, Heatmap visualizing expression of top-enriched genes in clusters of the grafted cells.
c-g, Expression of markers visualized on the t-SNE plot. The colors indicate the RPKM values.

Supplementary Figure 5

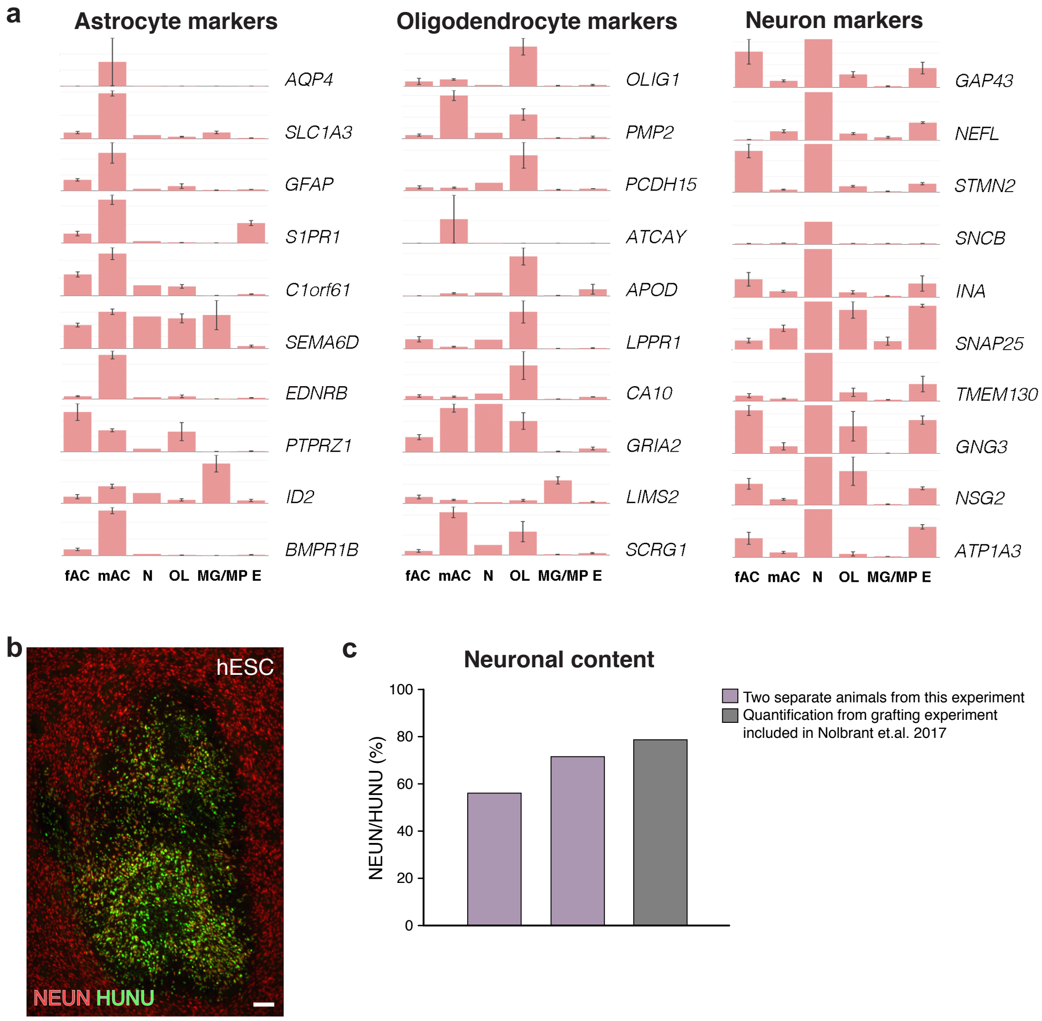

Supplementary Figure 5. **Validating cluster assignments and quantification of neurons**
a, Analysis of expression of top-enriched genes from clusters of the grafted cells in the RNA-seq transcriptome and splicing database (www.brainrnaseq.org). fAC – fetal astrocytes, mAC – mature astrocytes, N – neurons, OL – oligodendrocytes, MG– microglia, MP – macrophage, E – endothelial.
b, Representative HUNU/NEUN double immunostaining in hESC-derived grafted cells. Scale bar, 100 μM
c, Quantification of the HUNU/NEUN double positive cells from 2 hESC-grafted animals in this experiment and 1 animal from a previous experiment[1]. All cells in two sections (graft core and graft edge) per animal were quantified.

Supplementary Figure 6

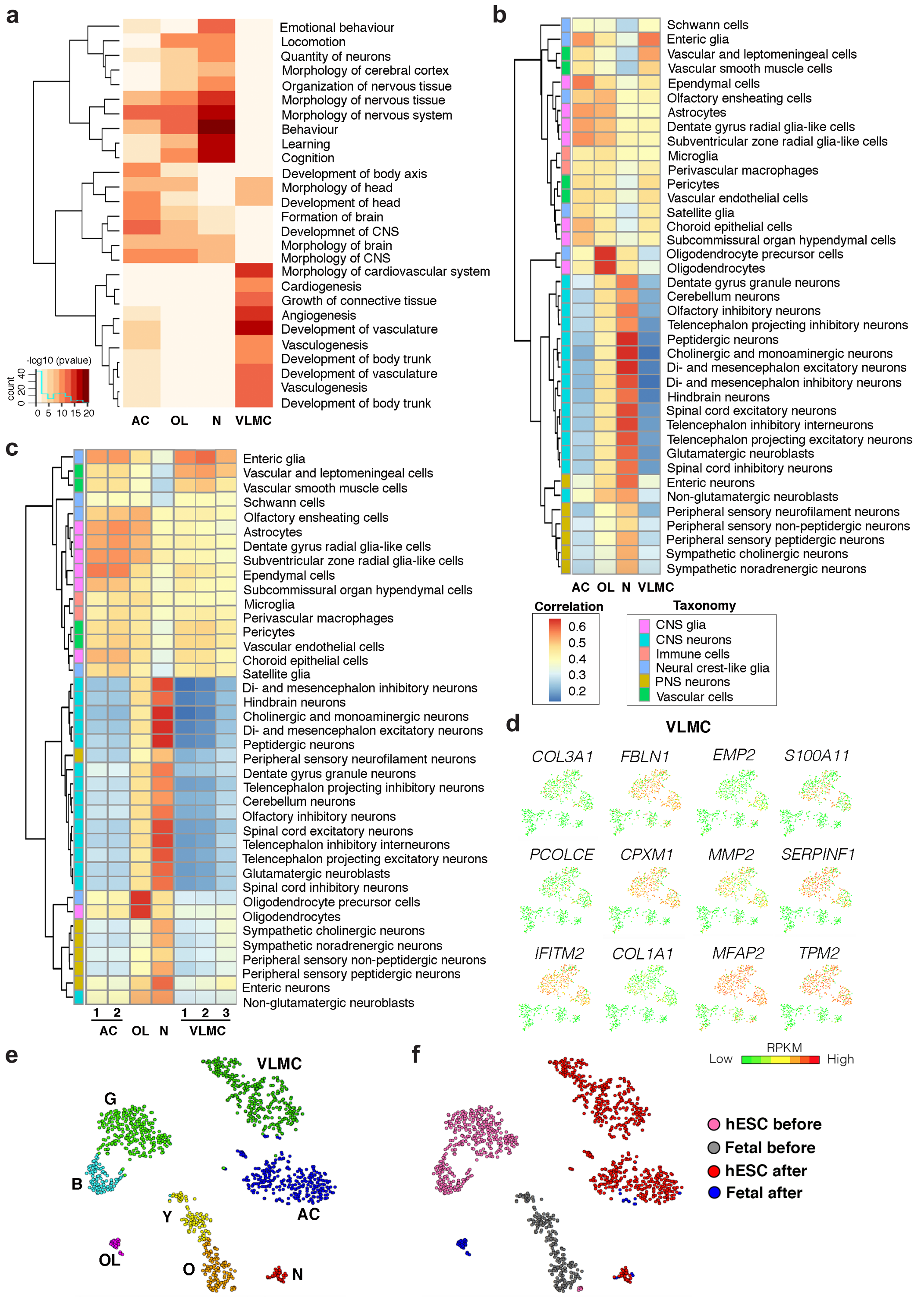

Supplementary Figure 6. Analysis of VLMC functional pathways and comparison with brain atlas

a, Top-significant Physiological System Development and Function categories from clusters of the grafted cells (Fig. 2a) as analyzed by Ingenuity Pathway Analysis (QIAGEN Inc., https://www.qiagenbioinformatics.com/products/ingenuity-pathway- analysis).

b-c, Correlation of clusters after grafting (horizontally) and mouse brain atlas (www.mousebrain.org) (vertically) as described in Methods.
d, Expression of VLMC markers visualized on the t-SNE plot of cells before grafting. The colors indicate the RPKM values.
e, t-SNE showing clustering of 1406 analyzed cells (660 before grafting [404 cells of hESC origin, 256 cells of fetal origin]; 746 after grafting [683 cells of hESC origin, 63 cells of fetal origin]). Cell type assignments: G = green cluster, B = blue cluster, O = orange cluster, Y = yellow cluster, OL = Oligodendrocyte, AC = Astrocyte, N = Neuron, VLMC.
f, Same t-SNE as in e) but with origin of cells: pink circles (hESCs before grafting), grey circles (fetal cells before grafting), red circles (hESCs after grafting) and blue circles (fetal cells after grafting).

Supplementary Figure 7

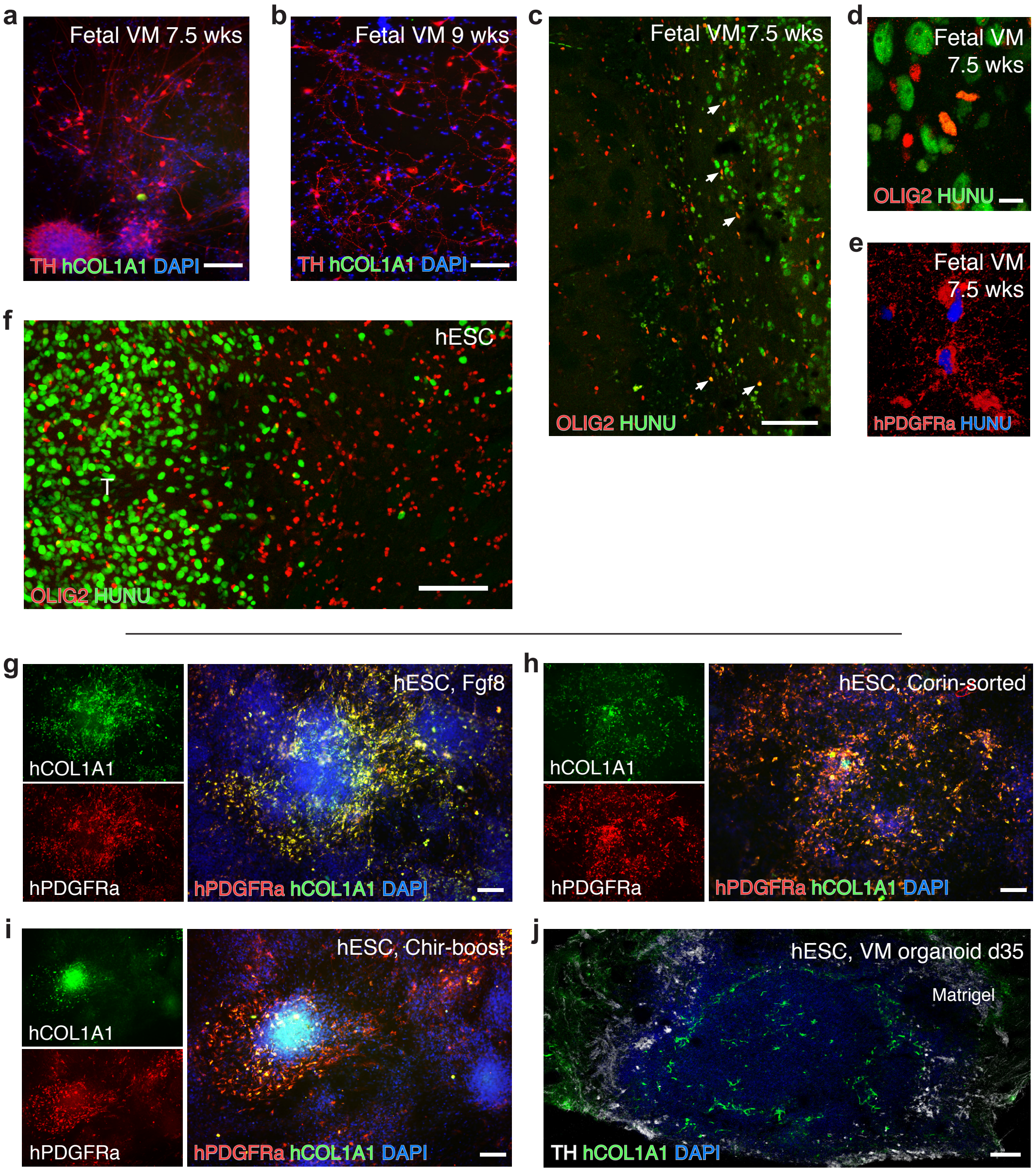

Supplementary Figure 7. **Validation of VLMCs in grafts and cultures**
a-b, Fetal cultures dissected from 7.5 week (a) or 9 week (b) old VM tissue double immunostained for TH/ hCOL1A1.
c-e, Fetal grafts double immunostained for HUNU/OLIG2 (c, d) and hPDGFRα/HUNU (e).
f, hESC-derived grafts double immunostained for OLIG2/HUNU.
g-i, Representative pictures of PDGFRα/hCOL1A1 double immunostaining in terminally differentiated hESC *in vitro* cultures derived by three different clinically relevant VM-patterning differentiation protocols: the protocol used in this study (g), a
protocol developed by the Takahashi lab where the cells are sorted based on CORIN prior to grafting (h)[2, 3] and a protocol developed in the Studer lab that uses CHIR boost instead of FGF8 for proper caudalization (https://patents.justia.com/patent/20180094242) (i). Nuclei were counterstained with DAPI.
j, TH/hCOL1A1 double immunostaining in self-organized midbrain patterned organoids.
Scale bars, 100 µM (a-c, f-j), 10 µM (d, e). T=transplant

Supplementary Figure 8

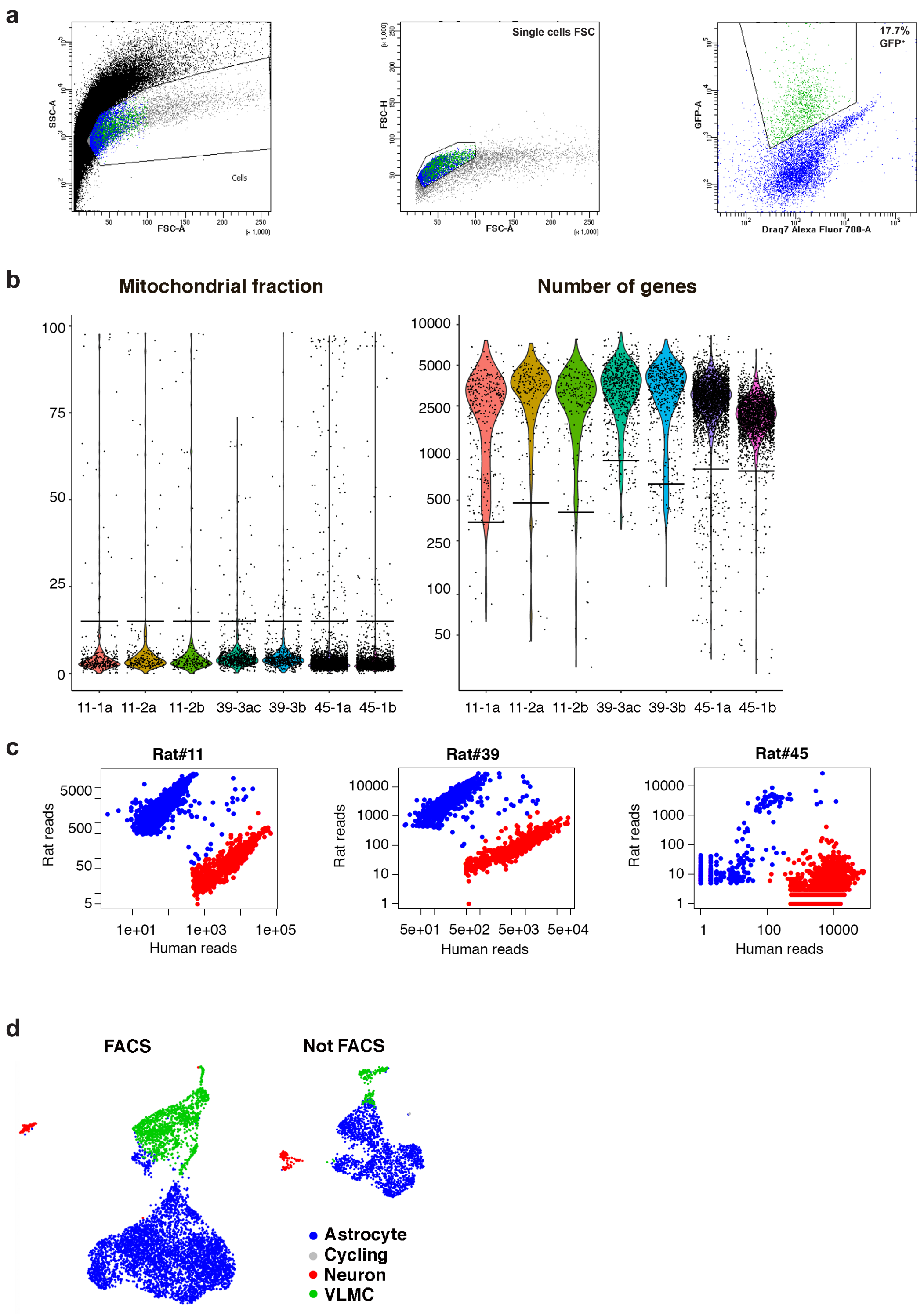

Supplementary Figure 8. **Isolation of midbrain grafted cells by FACS and quality control of sequenced cells**
a, FACS strategy for sorting of the single cells based on GFP expression.
b, Filtering of low quality cells based on mitochondrial fraction and number of detected genes per cell.
c, Filtering of cells based on alignment to the rat genome.
d, UMAP showing separate clustering of FACS sorted (n=5958) and not FACS sorted midbrain grafted cells (n=1917).

Supplementary Figure 9

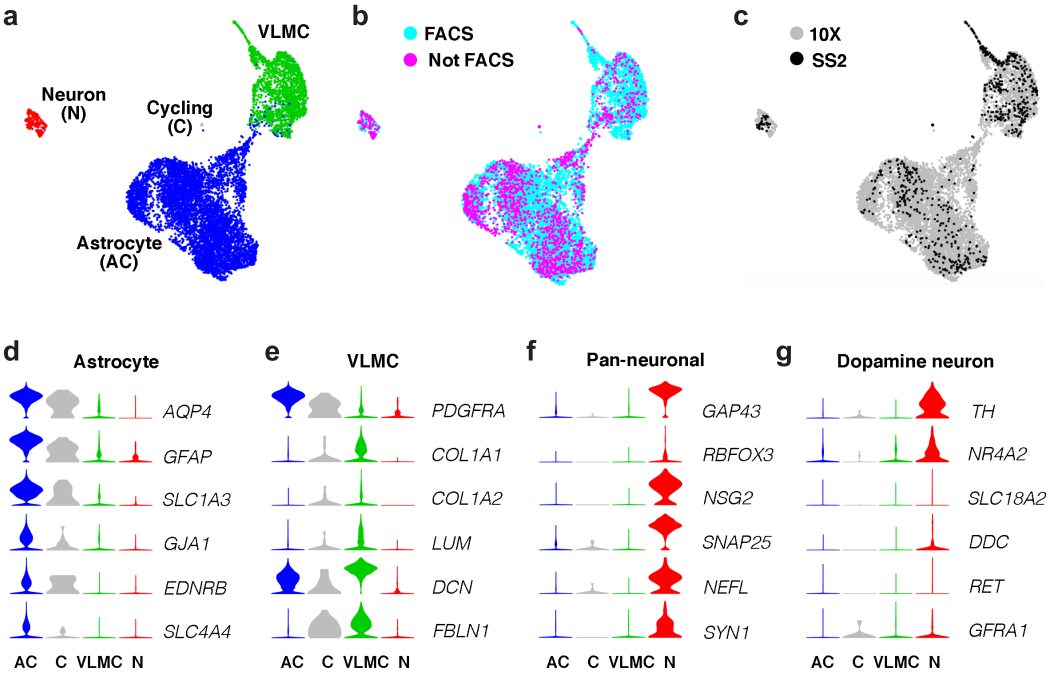

Supplementary Figure 9. **Integrated data analysis of grafted cells into the striatum and midbrain**
a-c, UMAP showing clustering (a), sorting (b) and type of library preparation and sequencing (c) of 8558 cells grafted into the striatum (683 cells, grafted rats n=2) and midbrain (7875 cells, grafted rats n=3).
d-g, Expression level per cluster for indicated genes. All indicated genes are enriched and known markers for astrocytes, VLMCs pan-neuronal cells and dopaminergic neurons.

Supplementary Figure 10

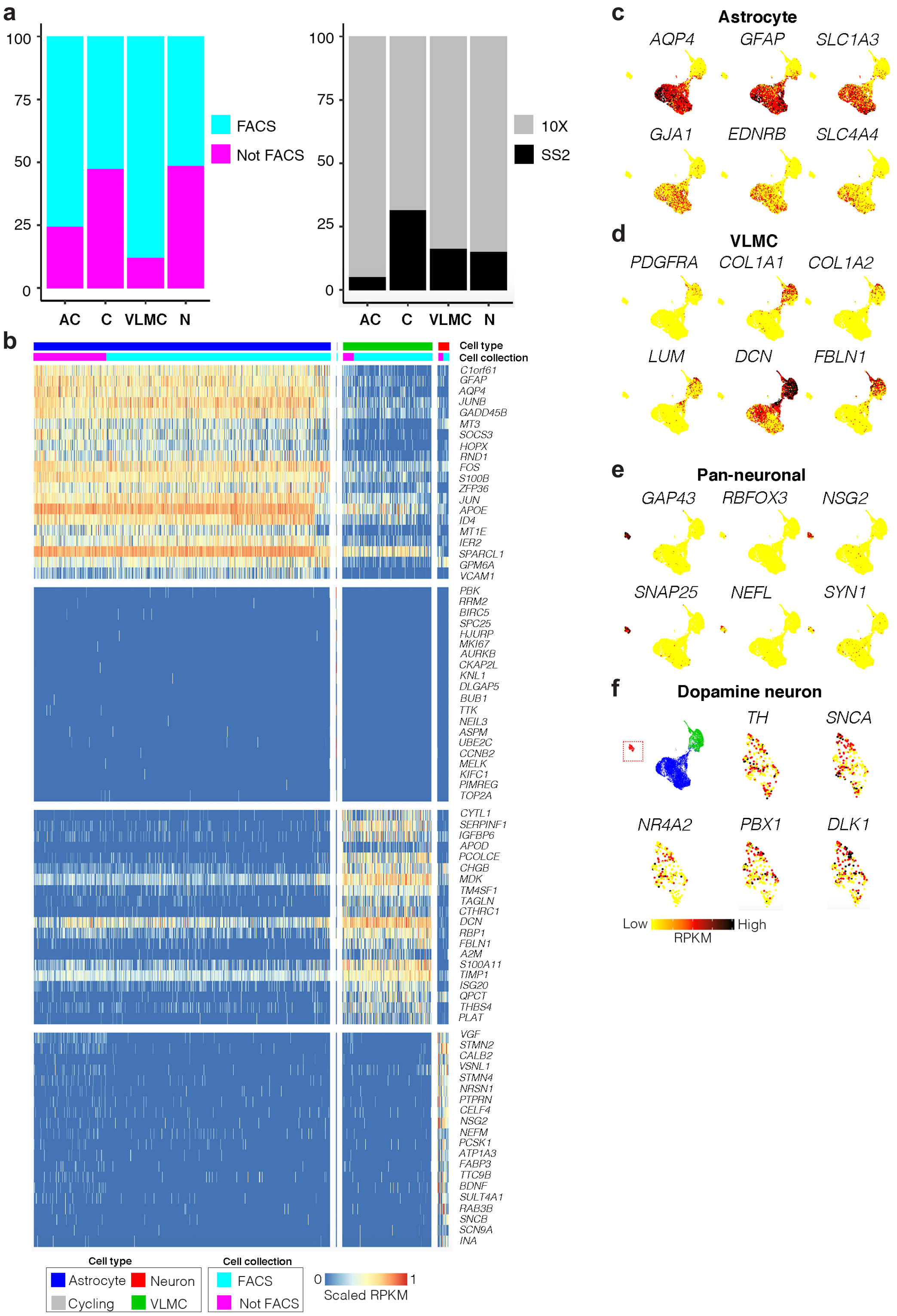

Supplementary Figure 10. **Analysis of scRNA-seq data from grafted cells into the striatum and midbrain**

a, Graphs representing proportion (%) of cell types (AC, C, VLMC, N) per sorting (FACS, Not FACS) and type of library preparation and sequencing (10X, SS2).

b, Heatmap visualizing expression of top-enriched genes in clusters of the grafted cells.

c-e, Expression of markers visualized on the UMAP plot. The colors indicate the RPKM values.

f, Expression of markers in the neuron cluster (cells marked in red) visualized on the UMAP plot. The colors indicate the RPKM values.

Supplementary Figure 11

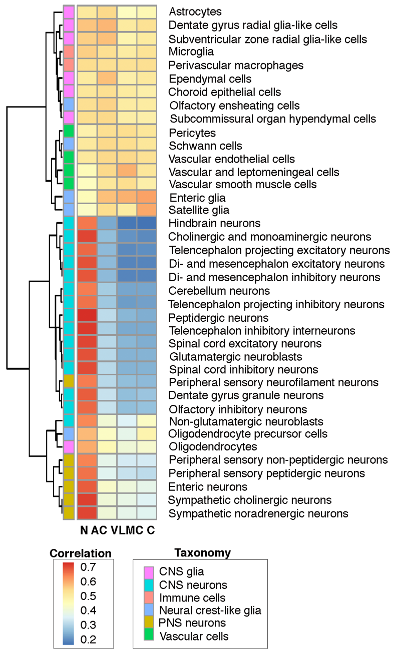

Supplementary Figure 11. **Comparison of clusters from striatal and midbrain hESC-derived grafts with brain atlas**

Correlation of clusters after grafting into striatum and midbrain (horizontally) and mouse brain atlas (www.mousebrain.org) (vertically) as described in Methods.

Supplementary Table 1. Antibodies

Antibodies				
**Name**	**Company**	**Product number**	**Lot Number**	**Dilution used**
HuNu	Merck Millipore	MAB1281, clone 235-1	3065575	1:100
TH	Merck Millipore	AB152	3086652	1:1000
NeuN	Merck Millipore	ABN78	2672962	1:500
hCOL1A1	R&D Systems	AF6220	CFJF0117091	1:200
COL1A1	Abcam	ab34710	GR3217975-4	1:200
hPDGFRα	Cell Signaling	5241	4	1:300
Olig2	Neuromics	RA25081	402230	1:500
GFP	Abcam	ab13970	GR3190550-4	1:1000
hNCAM	Santa Cruz Biotechnology	sc-106	I2217	1:1000

Supplementary References

1. Nolbrant, S., Heuer, A., Parmar, M. & Kirkeby, A. Generation of high-purity human ventral midbrain dopaminergic progenitors for in vitro maturation and intracerebral transplantation. *Nat Protoc* **12,** 1962–1979 (2017).
2. Doi, D. *et al.* Isolation of human induced pluripotent stem cell-derived dopaminergic progenitors by cell sorting for successful transplantation. *Stem Cell Reports* **2,** 337–350 (2014).
3. Kikuchi, T. *et al.* Human iPS cell-derived dopaminergic neurons function in a primate Parkinson's disease model. *Nature Publishing Group* **548,** 592–596 (2017).
