# Supplementary material for: Single cell transcriptomics identifies stem cell-derived graft composition in a model of Parkinson’s disease
Source: Nat Commun. 2020 May 15;11:2434. doi: 10.1038/s41467-020-16225-5 (PMC7229159; doi:10.1038/s41467-020-16225-5)
Supplement: Supplementary file 3 — Description of Additional Supplementary Files [file 41467_2020_16225_MOESM3_ESM.pdf]

Description of Additional Supplementary Files

File Name: Supplementary Data 1
Description: Gene list of clusters before grafting

File Name: Supplementary Data 2
Description: Gene list of clusters after grafting (grafted cells into the striatum)

File Name: Supplementary Data 3
Description: Gene enrichment analysis of clusters after grafting (grafted cells into the striatum)

File Name: Supplementary Data 4
Description: Gene list of clusters after grafting (grafted cells into the midbrain)

File Name: Supplementary Data 5
Description: Gene list of clusters after grafting (integrated data)
